# Supplementary figures and images for: Patterns in schizomid flagellum shape from elliptical Fourier analysis (part 2 of 5)
Source: Sci Rep. 2022 Mar 10;12:3896. doi: 10.1038/s41598-022-07823-y (PMC8913634; doi:10.1038/s41598-022-07823-y)

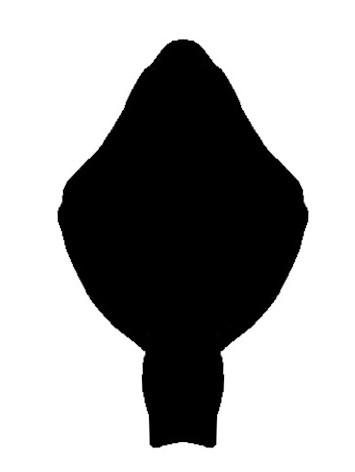

Supplement: Supplementary file 3 — Supplementary Information 3. [file 41598_2022_7823_MOESM3_ESM.zip › Han_vanderdrifti.jpg]

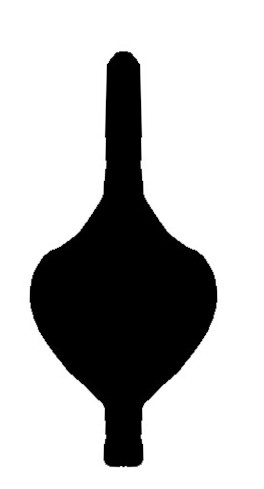

Supplement: Supplementary file 3 — Supplementary Information 3. [file 41598_2022_7823_MOESM3_ESM.zip › Han_yolandae.jpg]

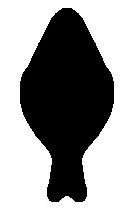

Supplement: Supplementary file 3 — Supplementary Information 3. [file 41598_2022_7823_MOESM3_ESM.zip › Har_contrerasi.jpg]

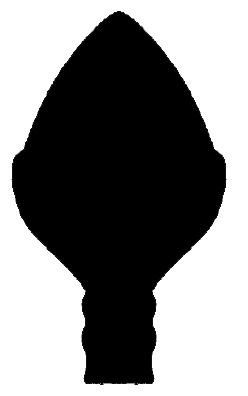

Supplement: Supplementary file 3 — Supplementary Information 3. [file 41598_2022_7823_MOESM3_ESM.zip › Har_mexicanus.jpg]

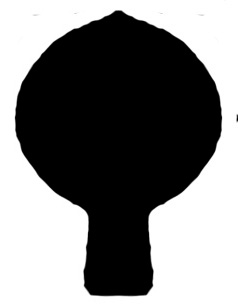

Supplement: Supplementary file 3 — Supplementary Information 3. [file 41598_2022_7823_MOESM3_ESM.zip › Har_mulaiki.jpg]

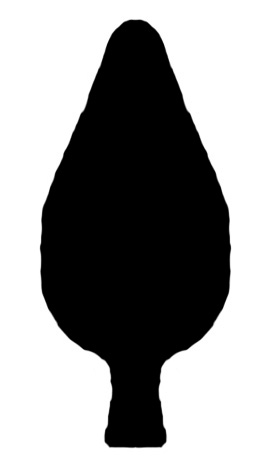

Supplement: Supplementary file 3 — Supplementary Information 3. [file 41598_2022_7823_MOESM3_ESM.zip › Har_reddelli.jpg]

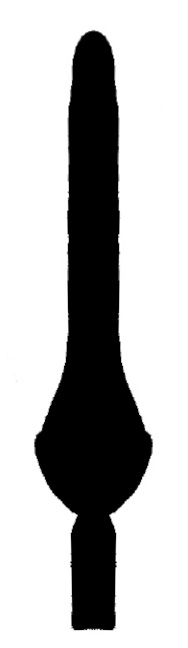

Supplement: Supplementary file 3 — Supplementary Information 3. [file 41598_2022_7823_MOESM3_ESM.zip › Het_goodnightorum.jpg]

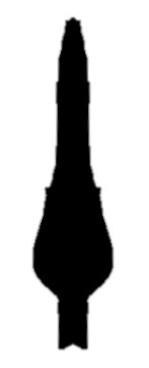

Supplement: Supplementary file 3 — Supplementary Information 3. [file 41598_2022_7823_MOESM3_ESM.zip › Het_kekchi.jpg]

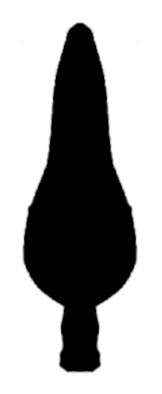

Supplement: Supplementary file 3 — Supplementary Information 3. [file 41598_2022_7823_MOESM3_ESM.zip › Het_meambar.jpg]

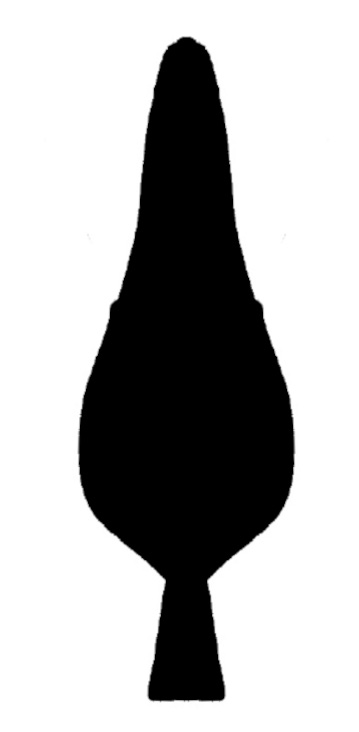

Supplement: Supplementary file 3 — Supplementary Information 3. [file 41598_2022_7823_MOESM3_ESM.zip › Het_orthoplax.jpg]

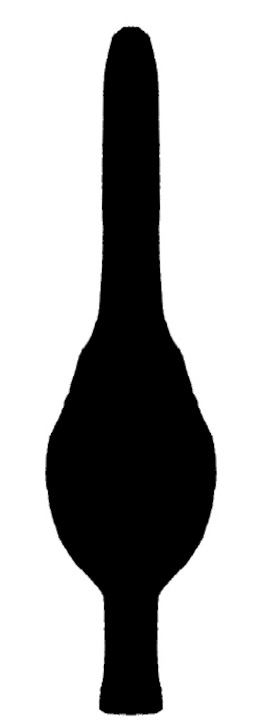

Supplement: Supplementary file 3 — Supplementary Information 3. [file 41598_2022_7823_MOESM3_ESM.zip › Het_silvino.jpg]

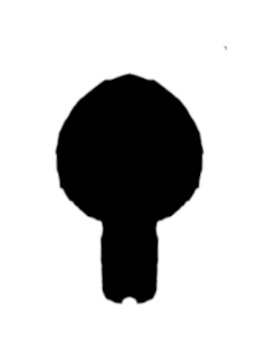

Supplement: Supplementary file 3 — Supplementary Information 3. [file 41598_2022_7823_MOESM3_ESM.zip › Htc_sierramaestrae.jpg]

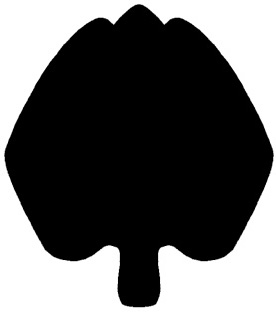

Supplement: Supplementary file 3 — Supplementary Information 3. [file 41598_2022_7823_MOESM3_ESM.zip › Hub_belkini.jpg]

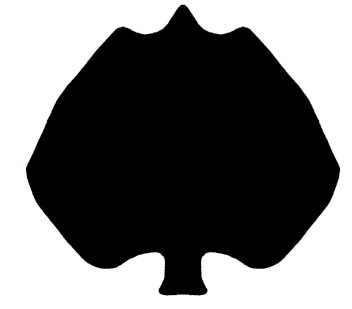

Supplement: Supplementary file 3 — Supplementary Information 3. [file 41598_2022_7823_MOESM3_ESM.zip › Hub_joshuaensis.jpg]

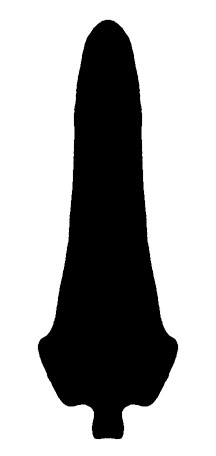

Supplement: Supplementary file 3 — Supplementary Information 3. [file 41598_2022_7823_MOESM3_ESM.zip › Hub_pentapeltis.jpg]

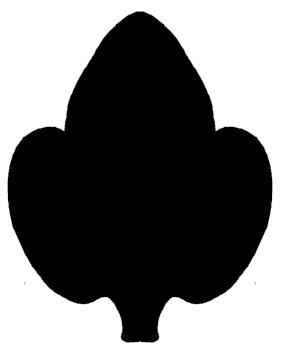

Supplement: Supplementary file 3 — Supplementary Information 3. [file 41598_2022_7823_MOESM3_ESM.zip › Hub_wessoni.jpg]

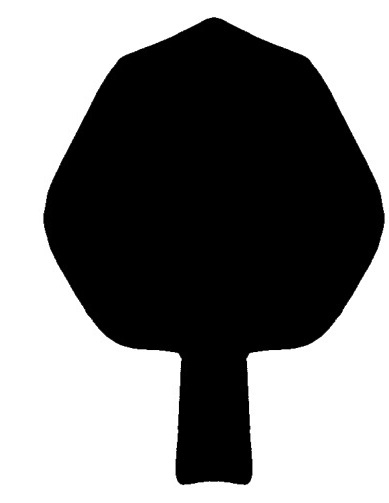

Supplement: Supplementary file 3 — Supplementary Information 3. [file 41598_2022_7823_MOESM3_ESM.zip › Jul_cooloola.jpg]

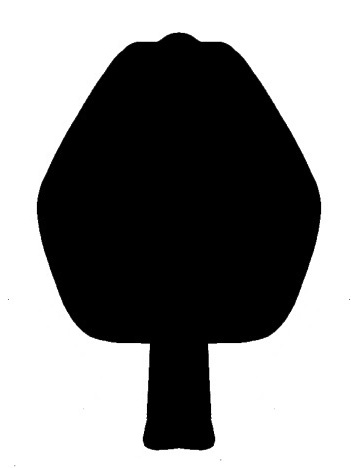

Supplement: Supplementary file 3 — Supplementary Information 3. [file 41598_2022_7823_MOESM3_ESM.zip › Jul_lawrencei.jpg]

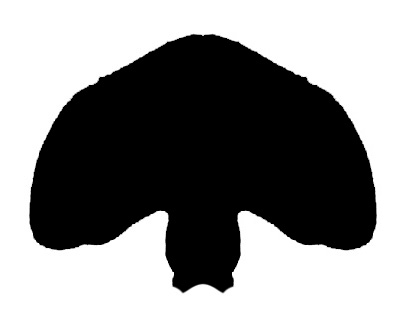

Supplement: Supplementary file 3 — Supplementary Information 3. [file 41598_2022_7823_MOESM3_ESM.zip › Ken_pekkai.jpg]

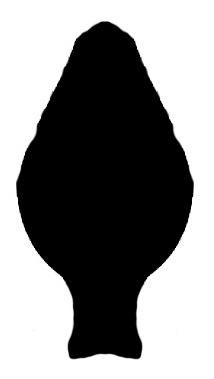

Supplement: Supplementary file 3 — Supplementary Information 3. [file 41598_2022_7823_MOESM3_ESM.zip › Law_atlanticus.jpg]

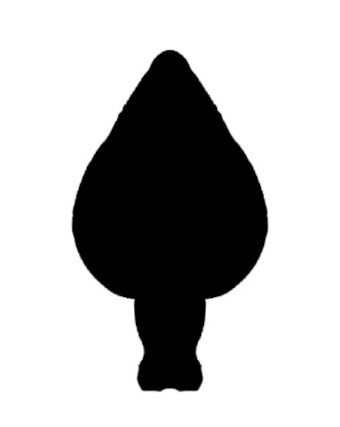

Supplement: Supplementary file 3 — Supplementary Information 3. [file 41598_2022_7823_MOESM3_ESM.zip › Law_bong.jpg]

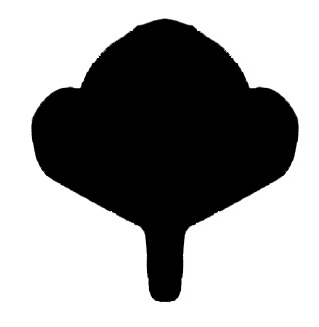

Supplement: Supplementary file 3 — Supplementary Information 3. [file 41598_2022_7823_MOESM3_ESM.zip › Lui_yunquensis.jpg]

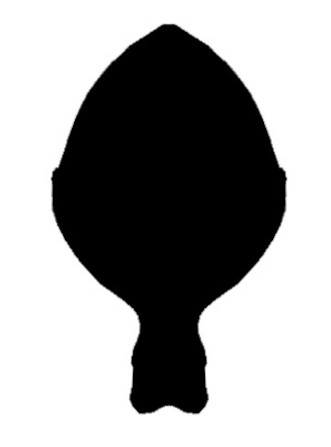

Supplement: Supplementary file 3 — Supplementary Information 3. [file 41598_2022_7823_MOESM3_ESM.zip › May_aluxe.jpg]

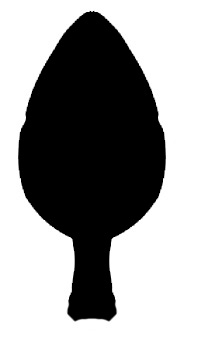

Supplement: Supplementary file 3 — Supplementary Information 3. [file 41598_2022_7823_MOESM3_ESM.zip › May_estorAV10.jpg]

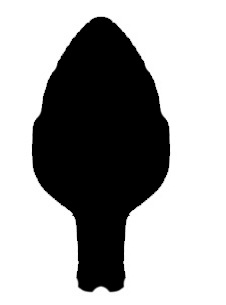

Supplement: Supplementary file 3 — Supplementary Information 3. [file 41598_2022_7823_MOESM3_ESM.zip › May_estorMR16.jpg]

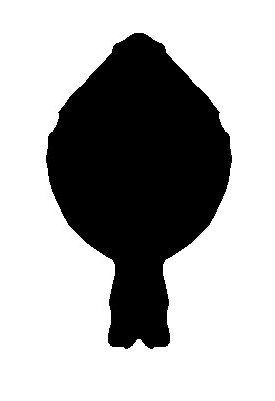

Supplement: Supplementary file 3 — Supplementary Information 3. [file 41598_2022_7823_MOESM3_ESM.zip › May_hoffmannae.jpg]

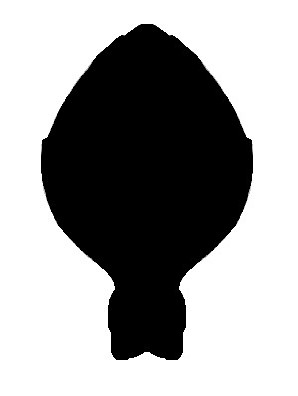

Supplement: Supplementary file 3 — Supplementary Information 3. [file 41598_2022_7823_MOESM3_ESM.zip › May_infernalis.jpg]

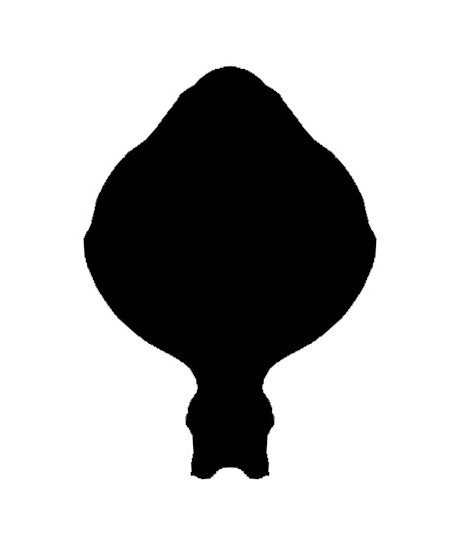

Supplement: Supplementary file 3 — Supplementary Information 3. [file 41598_2022_7823_MOESM3_ESM.zip › May_kaamuul.jpg]

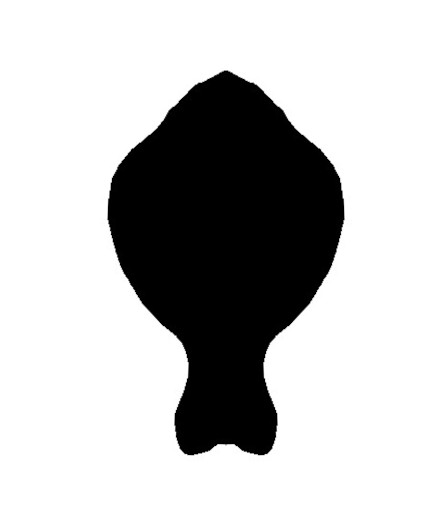

Supplement: Supplementary file 3 — Supplementary Information 3. [file 41598_2022_7823_MOESM3_ESM.zip › May_loobil.jpg]

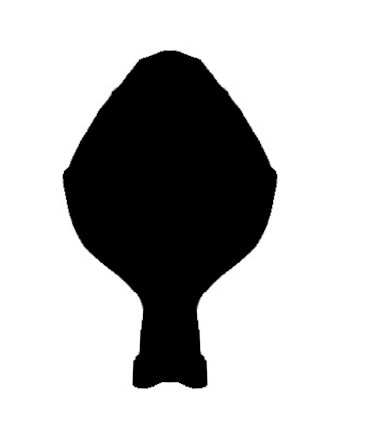

Supplement: Supplementary file 3 — Supplementary Information 3. [file 41598_2022_7823_MOESM3_ESM.zip › May_tzotzil.jpg]

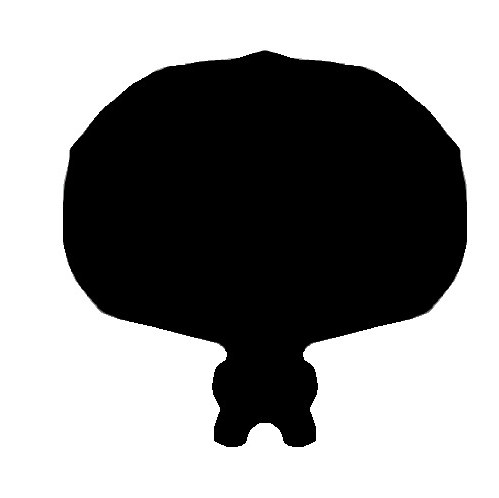

Supplement: Supplementary file 3 — Supplementary Information 3. [file 41598_2022_7823_MOESM3_ESM.zip › May_yaax.jpg]

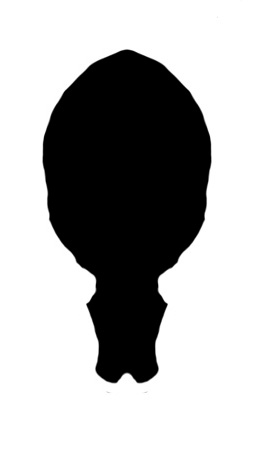

Supplement: Supplementary file 3 — Supplementary Information 3. [file 41598_2022_7823_MOESM3_ESM.zip › Meg_mossambicus.jpg]

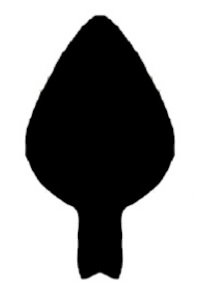

Supplement: Supplementary file 3 — Supplementary Information 3. [file 41598_2022_7823_MOESM3_ESM.zip › Nad_carajas.jpg]

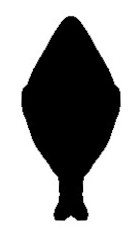

Supplement: Supplementary file 3 — Supplementary Information 3. [file 41598_2022_7823_MOESM3_ESM.zip › Nah_bokmai.JPG]

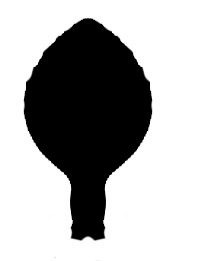

Supplement: Supplementary file 3 — Supplementary Information 3. [file 41598_2022_7823_MOESM3_ESM.zip › Nah_caballero.jpg]

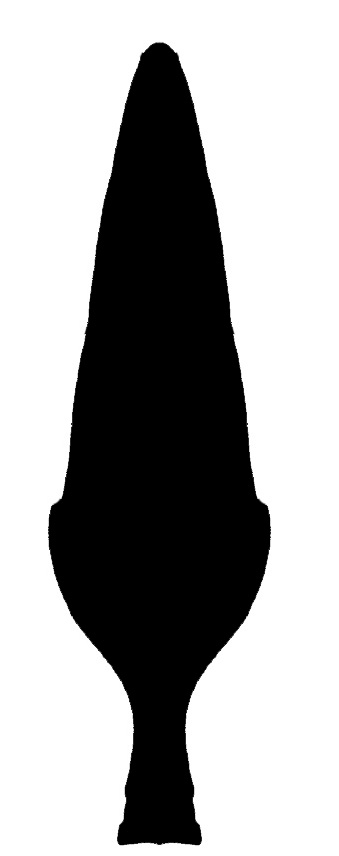

Supplement: Supplementary file 3 — Supplementary Information 3. [file 41598_2022_7823_MOESM3_ESM.zip › Nah_lanceolatus.jpg]

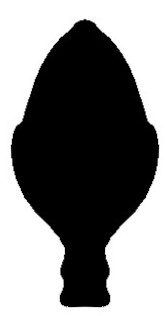

Supplement: Supplementary file 3 — Supplementary Information 3. [file 41598_2022_7823_MOESM3_ESM.zip › Nah_pallidus.jpg]

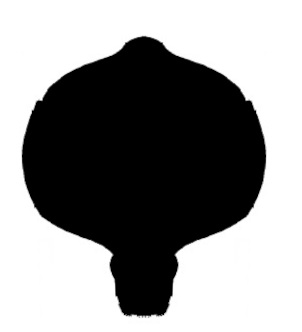

Supplement: Supplementary file 3 — Supplementary Information 3. [file 41598_2022_7823_MOESM3_ESM.zip › Neo_tikaderi.jpg]

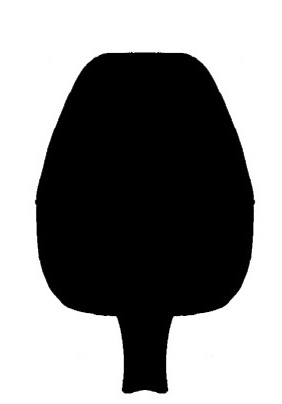

Supplement: Supplementary file 3 — Supplementary Information 3. [file 41598_2022_7823_MOESM3_ESM.zip › Not_aterpes.jpg]

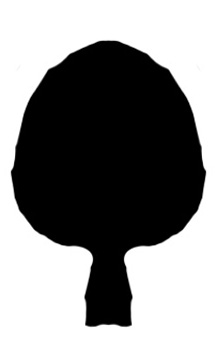

Supplement: Supplementary file 3 — Supplementary Information 3. [file 41598_2022_7823_MOESM3_ESM.zip › Not_bronwenae.jpg]

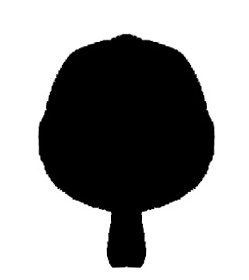

Supplement: Supplementary file 3 — Supplementary Information 3. [file 41598_2022_7823_MOESM3_ESM.zip › Not_curiosus.jpg]

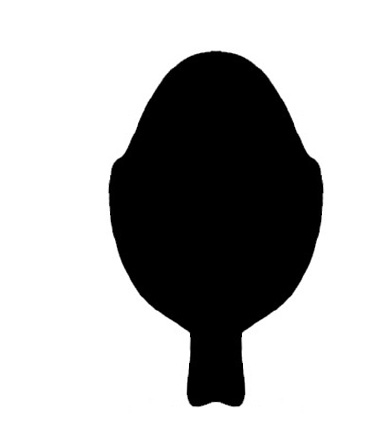

Supplement: Supplementary file 3 — Supplementary Information 3. [file 41598_2022_7823_MOESM3_ESM.zip › Not_daviesae.jpg]

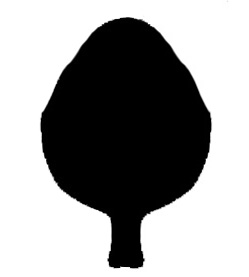

Supplement: Supplementary file 3 — Supplementary Information 3. [file 41598_2022_7823_MOESM3_ESM.zip › Not_ingham.jpg]

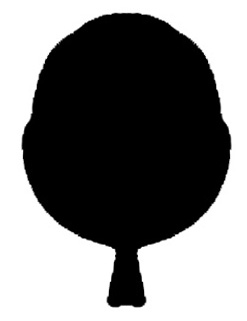

Supplement: Supplementary file 3 — Supplementary Information 3. [file 41598_2022_7823_MOESM3_ESM.zip › Not_jacquelinae.jpg]

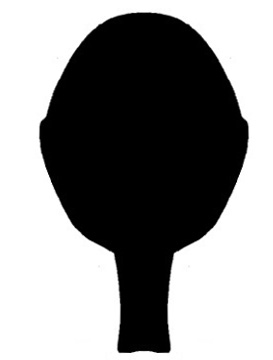

Supplement: Supplementary file 3 — Supplementary Information 3. [file 41598_2022_7823_MOESM3_ESM.zip › Not_ker.jpg]

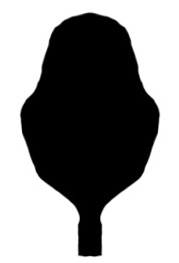

Supplement: Supplementary file 3 — Supplementary Information 3. [file 41598_2022_7823_MOESM3_ESM.zip › Not_majesticus.jpg]

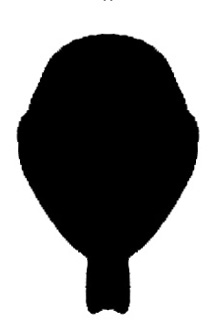

Supplement: Supplementary file 3 — Supplementary Information 3. [file 41598_2022_7823_MOESM3_ESM.zip › Not_maurophila.jpg]

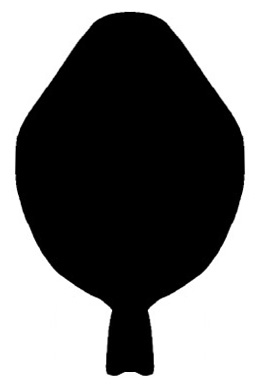

Supplement: Supplementary file 3 — Supplementary Information 3. [file 41598_2022_7823_MOESM3_ESM.zip › Not_rentzi.jpg]

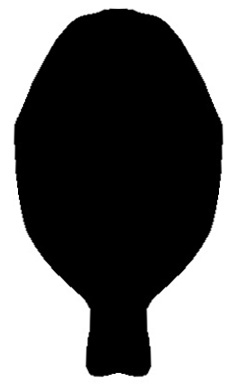

Supplement: Supplementary file 3 — Supplementary Information 3. [file 41598_2022_7823_MOESM3_ESM.zip › Not_spec.jpg]

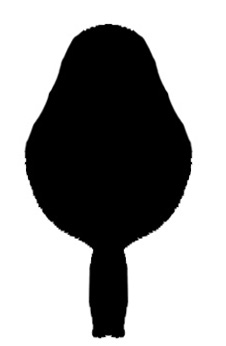

Supplement: Supplementary file 3 — Supplementary Information 3. [file 41598_2022_7823_MOESM3_ESM.zip › Not_wudjl.jpg]

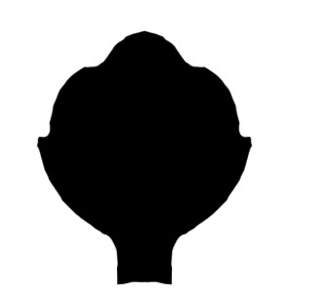

Supplement: Supplementary file 3 — Supplementary Information 3. [file 41598_2022_7823_MOESM3_ESM.zip › Ocu_biocellatus.jpg]

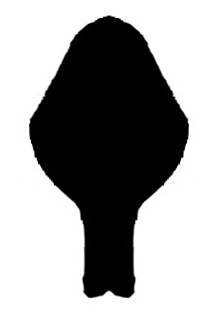

Supplement: Supplementary file 3 — Supplementary Information 3. [file 41598_2022_7823_MOESM3_ESM.zip › Olm_brujo.jpg]

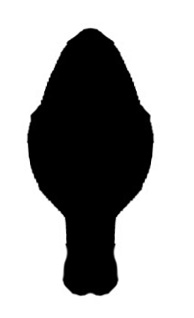

Supplement: Supplementary file 3 — Supplementary Information 3. [file 41598_2022_7823_MOESM3_ESM.zip › Olm_cruzlopezi.jpg]

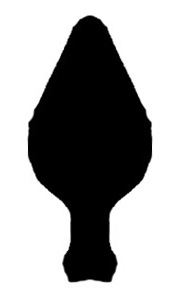

Supplement: Supplementary file 3 — Supplementary Information 3. [file 41598_2022_7823_MOESM3_ESM.zip › Olm_santibanezi.jpg]

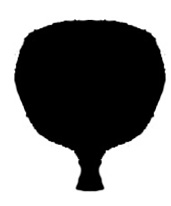

Supplement: Supplementary file 3 — Supplementary Information 3. [file 41598_2022_7823_MOESM3_ESM.zip › Ori_luzonicus.jpg]

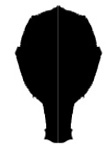

Supplement: Supplementary file 3 — Supplementary Information 3. [file 41598_2022_7823_MOESM3_ESM.zip › Ori_sawadai.jpg]

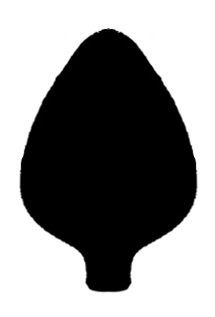

Supplement: Supplementary file 3 — Supplementary Information 3. [file 41598_2022_7823_MOESM3_ESM.zip › Ovo_lunatus.jpg]

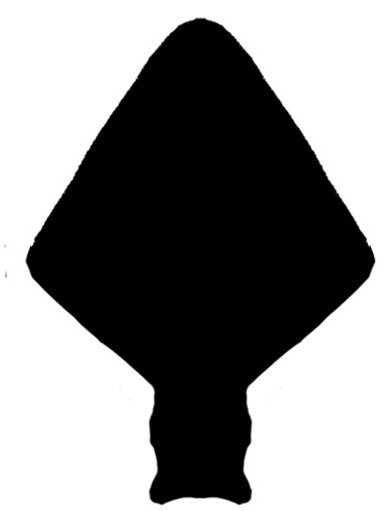

Supplement: Supplementary file 3 — Supplementary Information 3. [file 41598_2022_7823_MOESM3_ESM.zip › Pac_lacandonus.jpg]

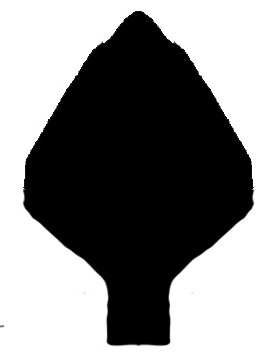

Supplement: Supplementary file 3 — Supplementary Information 3. [file 41598_2022_7823_MOESM3_ESM.zip › Pac_moisii.jpg]

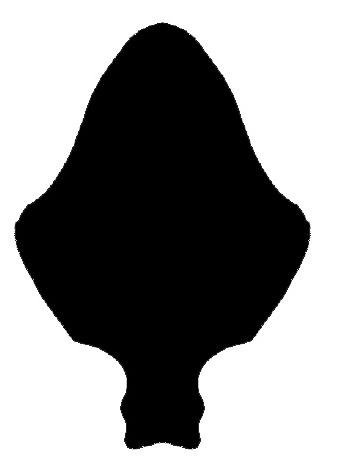

Supplement: Supplementary file 3 — Supplementary Information 3. [file 41598_2022_7823_MOESM3_ESM.zip › Pac_stewarti.jpg]

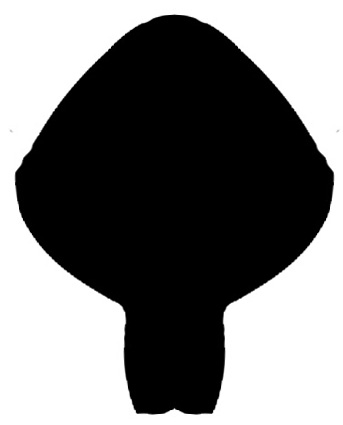

Supplement: Supplementary file 3 — Supplementary Information 3. [file 41598_2022_7823_MOESM3_ESM.zip › Pac_tepezcuintle.jpg]

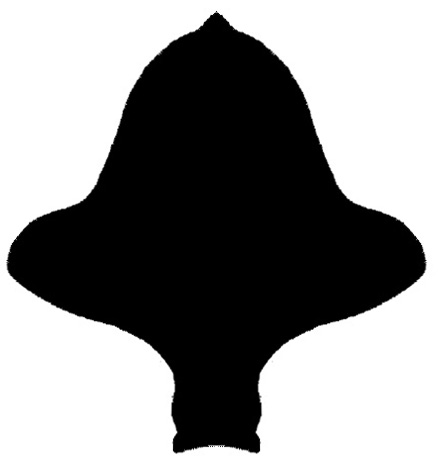

Supplement: Supplementary file 3 — Supplementary Information 3. [file 41598_2022_7823_MOESM3_ESM.zip › Pac_trilobatus.jpg]

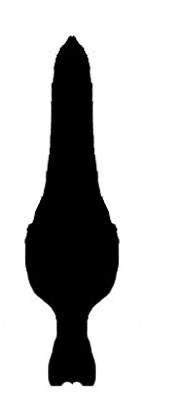

Supplement: Supplementary file 3 — Supplementary Information 3. [file 41598_2022_7823_MOESM3_ESM.zip › Pia_bacata.jpg]

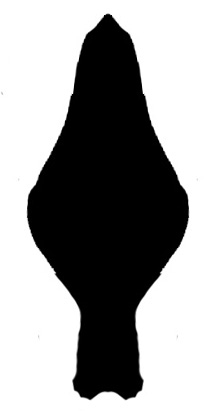

Supplement: Supplementary file 3 — Supplementary Information 3. [file 41598_2022_7823_MOESM3_ESM.zip › Pia_bijagua.jpg]

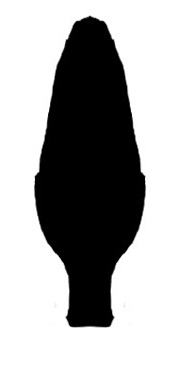

Supplement: Supplementary file 3 — Supplementary Information 3. [file 41598_2022_7823_MOESM3_ESM.zip › Pia_escalerete.jpg]

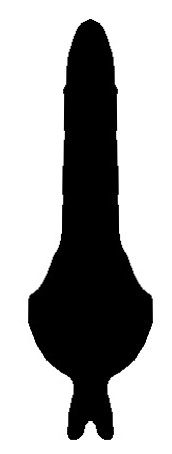

Supplement: Supplementary file 3 — Supplementary Information 3. [file 41598_2022_7823_MOESM3_ESM.zip › Pia_guipongai.jpg]

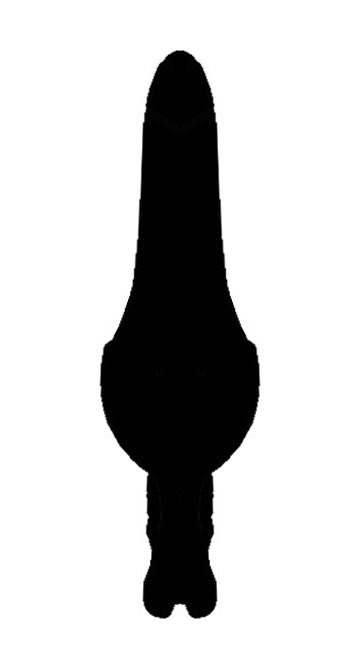

Supplement: Supplementary file 3 — Supplementary Information 3. [file 41598_2022_7823_MOESM3_ESM.zip › Pia_pioi.jpg]

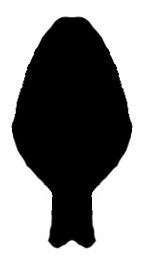

Supplement: Supplementary file 3 — Supplementary Information 3. [file 41598_2022_7823_MOESM3_ESM.zip › Pia_turbacoensis.jpg]

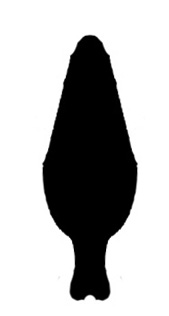

Supplement: Supplementary file 3 — Supplementary Information 3. [file 41598_2022_7823_MOESM3_ESM.zip › Pia_villarreali.jpg]

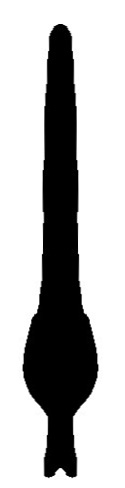

Supplement: Supplementary file 3 — Supplementary Information 3. [file 41598_2022_7823_MOESM3_ESM.zip › Pia_virichaj.jpg]

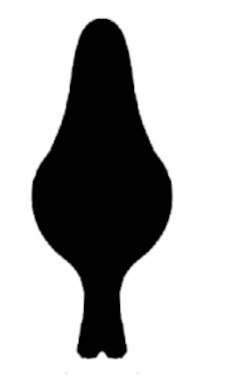

Supplement: Supplementary file 3 — Supplementary Information 3. [file 41598_2022_7823_MOESM3_ESM.zip › Pia_youngi.jpg]

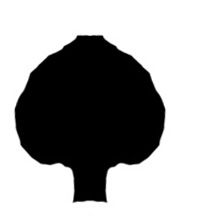

Supplement: Supplementary file 3 — Supplementary Information 3. [file 41598_2022_7823_MOESM3_ESM.zip › Pin_marmoreus.jpg]

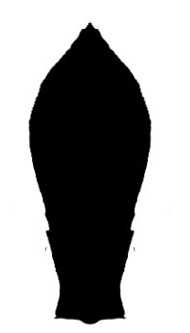

Supplement: Supplementary file 3 — Supplementary Information 3. [file 41598_2022_7823_MOESM3_ESM.zip › Pro_franckei.jpg]

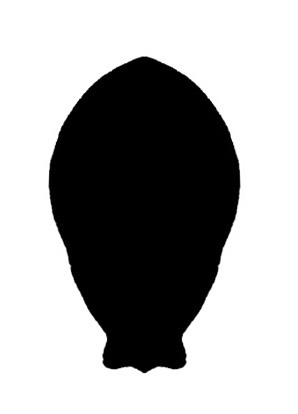

Supplement: Supplementary file 3 — Supplementary Information 3. [file 41598_2022_7823_MOESM3_ESM.zip › Pro_occidentalis.jpg]

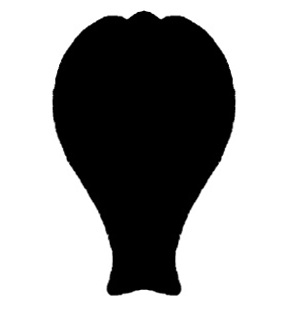

Supplement: Supplementary file 3 — Supplementary Information 3. [file 41598_2022_7823_MOESM3_ESM.zip › Pro_pachypalpus.jpg]

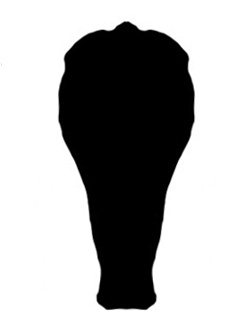

Supplement: Supplementary file 3 — Supplementary Information 3. [file 41598_2022_7823_MOESM3_ESM.zip › Pro_rowlandi.jpg]

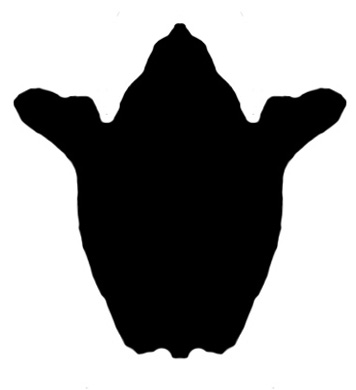

Supplement: Supplementary file 3 — Supplementary Information 3. [file 41598_2022_7823_MOESM3_ESM.zip › Pro_sprousei1.jpg]

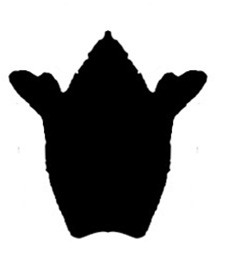

Supplement: Supplementary file 3 — Supplementary Information 3. [file 41598_2022_7823_MOESM3_ESM.zip › Pro_sprousei2.jpg]

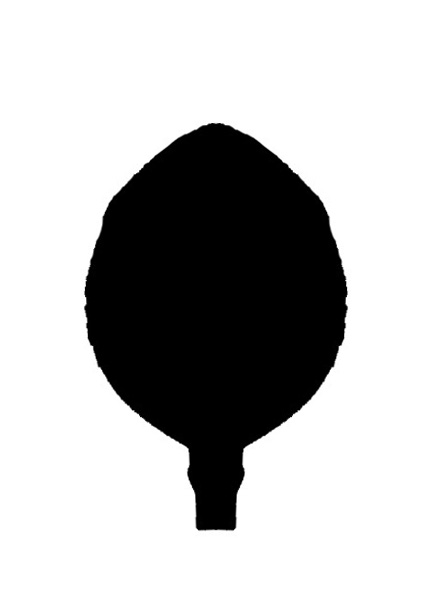

Supplement: Supplementary file 3 — Supplementary Information 3. [file 41598_2022_7823_MOESM3_ESM.zip › Red_cubensisArmas.jpg]

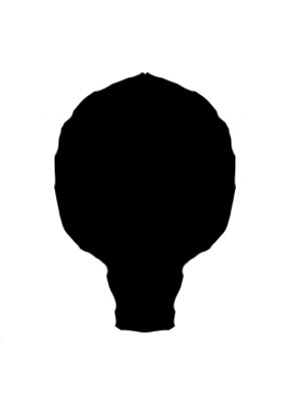

Supplement: Supplementary file 3 — Supplementary Information 3. [file 41598_2022_7823_MOESM3_ESM.zip › Red_cubensisTer.jpg]

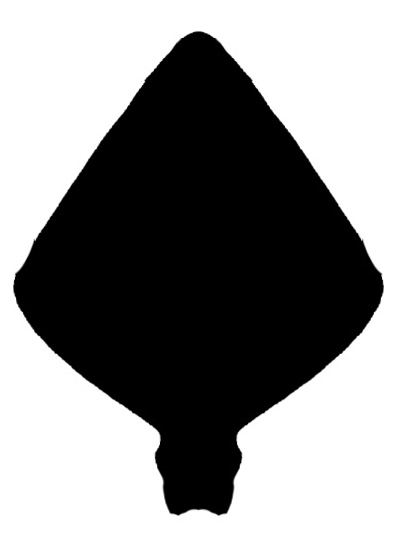

Supplement: Supplementary file 3 — Supplementary Information 3. [file 41598_2022_7823_MOESM3_ESM.zip › Row_abeli.jpg]

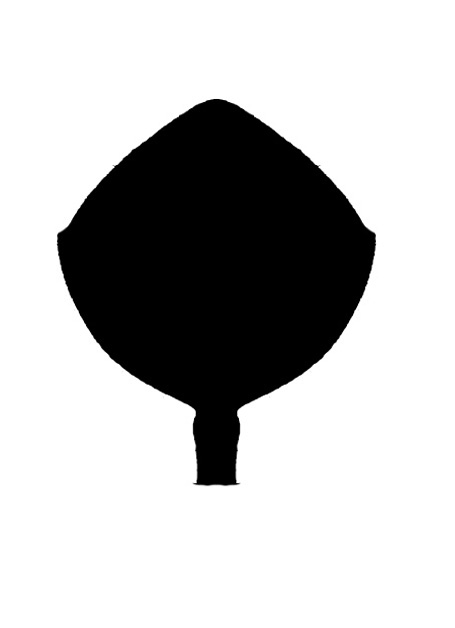

Supplement: Supplementary file 3 — Supplementary Information 3. [file 41598_2022_7823_MOESM3_ESM.zip › Row_anasilviae.jpg]

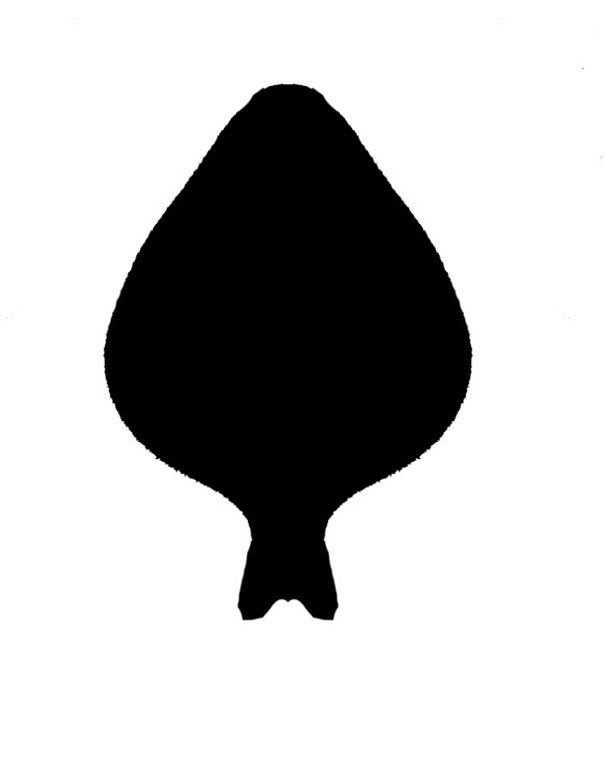

Supplement: Supplementary file 3 — Supplementary Information 3. [file 41598_2022_7823_MOESM3_ESM.zip › Row_arduus.jpg]

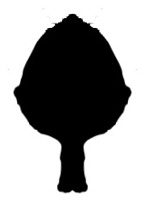

Supplement: Supplementary file 3 — Supplementary Information 3. [file 41598_2022_7823_MOESM3_ESM.zip › Row_arenicola.jpg]

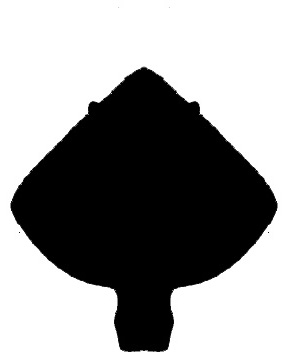

Supplement: Supplementary file 3 — Supplementary Information 3. [file 41598_2022_7823_MOESM3_ESM.zip › Row_biconourus.jpg]

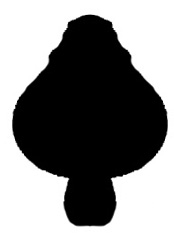

Supplement: Supplementary file 3 — Supplementary Information 3. [file 41598_2022_7823_MOESM3_ESM.zip › Row_candidae.jpg]

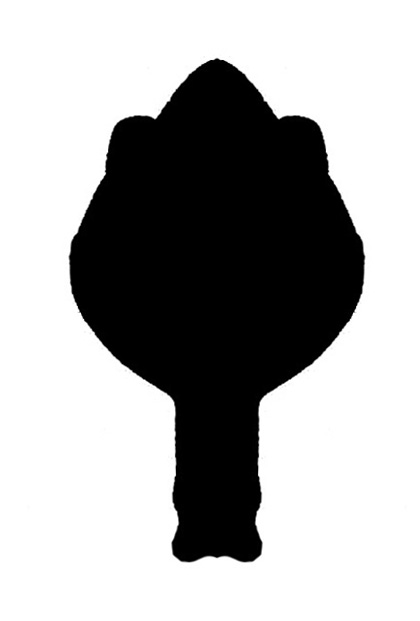

Supplement: Supplementary file 3 — Supplementary Information 3. [file 41598_2022_7823_MOESM3_ESM.zip › Row_casabito.jpg]

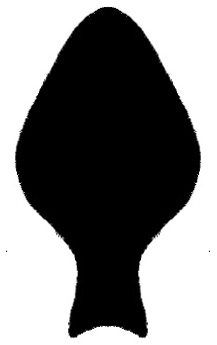

Supplement: Supplementary file 3 — Supplementary Information 3. [file 41598_2022_7823_MOESM3_ESM.zip › Row_cousinensis.jpg]

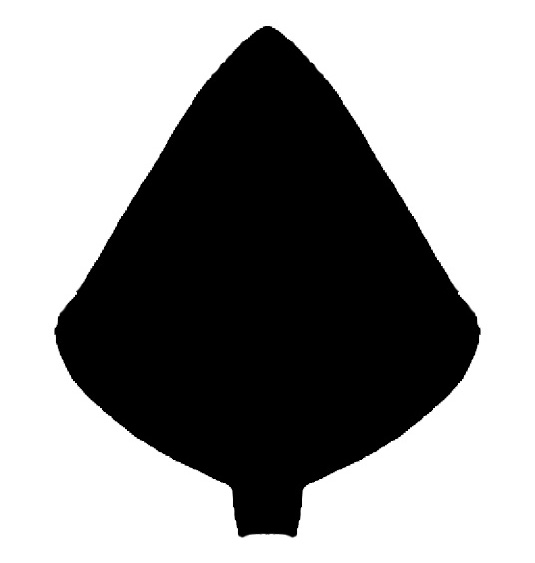

Supplement: Supplementary file 3 — Supplementary Information 3. [file 41598_2022_7823_MOESM3_ESM.zip › Row_cupeyalensis.jpg]

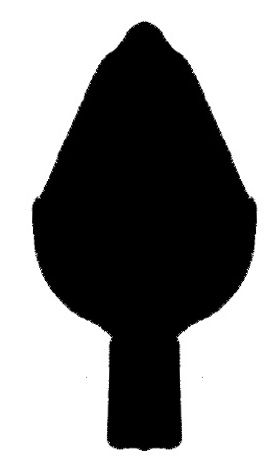

Supplement: Supplementary file 3 — Supplementary Information 3. [file 41598_2022_7823_MOESM3_ESM.zip › Row_desecheo.jpg]

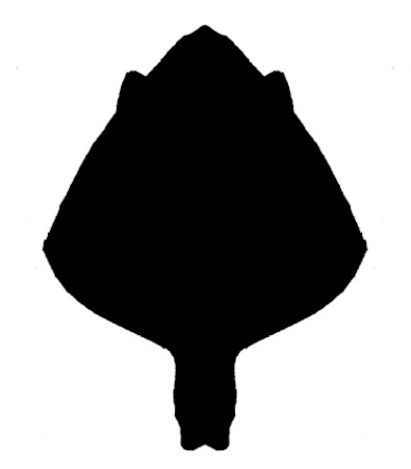

Supplement: Supplementary file 3 — Supplementary Information 3. [file 41598_2022_7823_MOESM3_ESM.zip › Row_ducoudrayi.jpg]

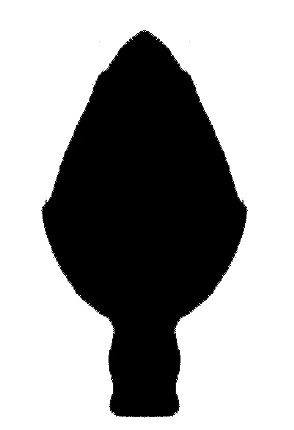

Supplement: Supplementary file 3 — Supplementary Information 3. [file 41598_2022_7823_MOESM3_ESM.zip › Row_dumitrescoae.jpg]

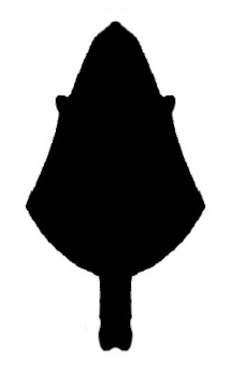

Supplement: Supplementary file 3 — Supplementary Information 3. [file 41598_2022_7823_MOESM3_ESM.zip › Row_engombe.jpg]

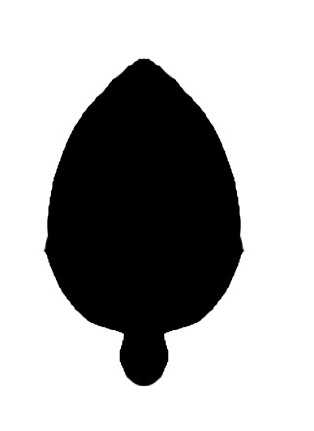

Supplement: Supplementary file 3 — Supplementary Information 3. [file 41598_2022_7823_MOESM3_ESM.zip › Row_falcifemur.jpg]

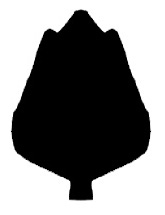

Supplement: Supplementary file 3 — Supplementary Information 3. [file 41598_2022_7823_MOESM3_ESM.zip › Row_florenciae.jpg]

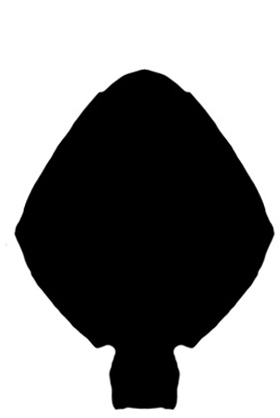

Supplement: Supplementary file 3 — Supplementary Information 3. [file 41598_2022_7823_MOESM3_ESM.zip › Row_gladiger.jpg]

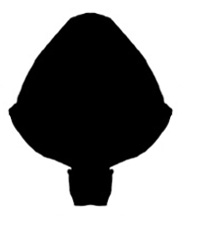

Supplement: Supplementary file 3 — Supplementary Information 3. [file 41598_2022_7823_MOESM3_ESM.zip › Row_gracilis.jpg]

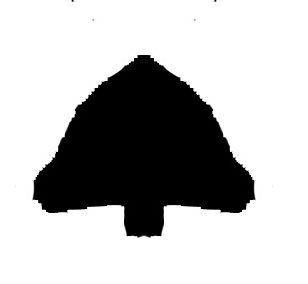

Supplement: Supplementary file 3 — Supplementary Information 3. [file 41598_2022_7823_MOESM3_ESM.zip › Row_guama.jpg]

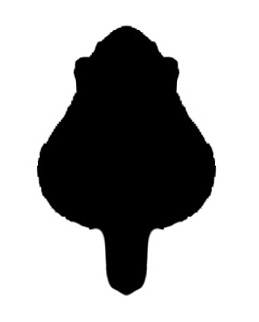

Supplement: Supplementary file 3 — Supplementary Information 3. [file 41598_2022_7823_MOESM3_ESM.zip › Row_guamuhaya.jpg]

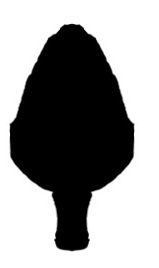

Supplement: Supplementary file 3 — Supplementary Information 3. [file 41598_2022_7823_MOESM3_ESM.zip › Row_guantanamero.jpg]
