# Supplementary figures and images for: Patterns in schizomid flagellum shape from elliptical Fourier analysis (part 1 of 5)
Source: Sci Rep. 2022 Mar 10;12:3896. doi: 10.1038/s41598-022-07823-y (PMC8913634; doi:10.1038/s41598-022-07823-y)

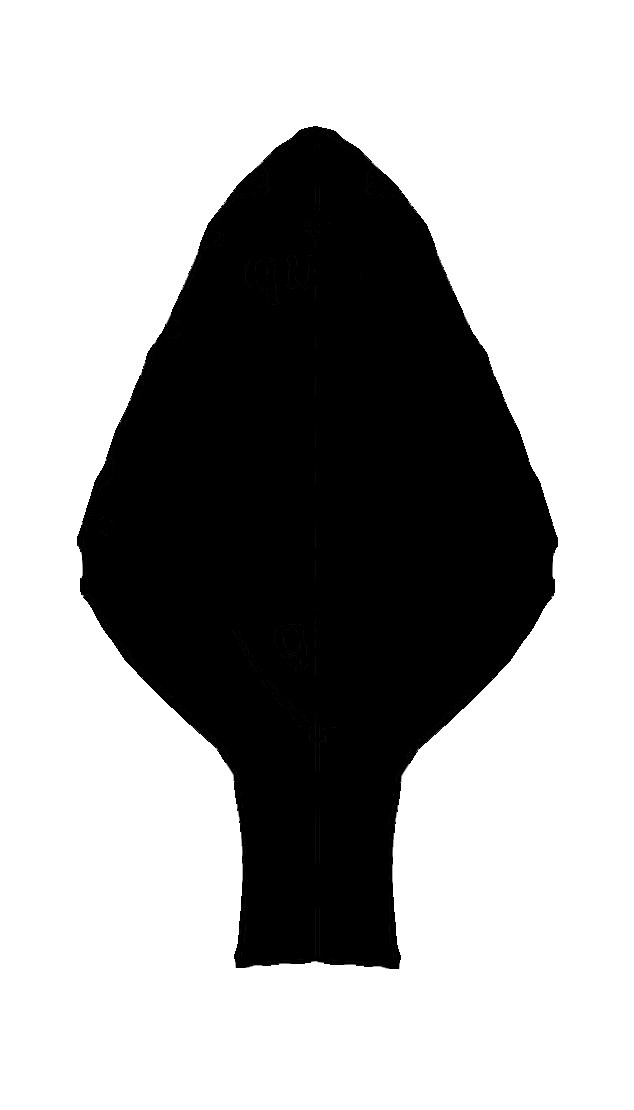

Supplement: Supplementary file 3 — Supplementary Information 3. [file 41598_2022_7823_MOESM3_ESM.zip › Adi_duckei.JPG]

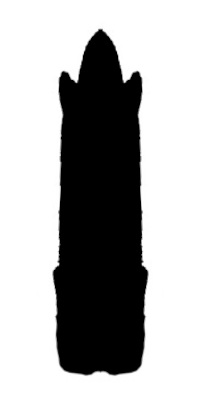

Supplement: Supplementary file 3 — Supplementary Information 3. [file 41598_2022_7823_MOESM3_ESM.zip › Aga_huitzmolotitlensis.jpg]

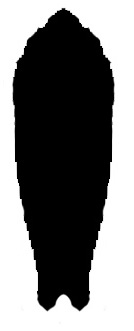

Supplement: Supplementary file 3 — Supplementary Information 3. [file 41598_2022_7823_MOESM3_ESM.zip › Aga_juxtlahuacensis.jpg]

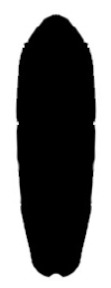

Supplement: Supplementary file 3 — Supplementary Information 3. [file 41598_2022_7823_MOESM3_ESM.zip › Aga_lucifer.jpg]

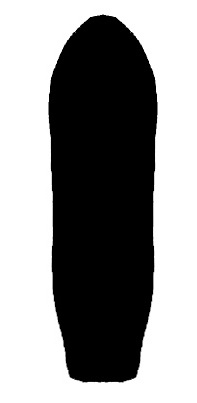

Supplement: Supplementary file 3 — Supplementary Information 3. [file 41598_2022_7823_MOESM3_ESM.zip › Aga_patei.jpg]

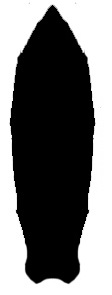

Supplement: Supplementary file 3 — Supplementary Information 3. [file 41598_2022_7823_MOESM3_ESM.zip › Aga_tamaulipensis.jpg]

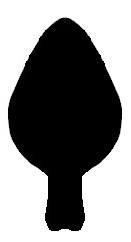

Supplement: Supplementary file 3 — Supplementary Information 3. [file 41598_2022_7823_MOESM3_ESM.zip › Amb_aquismon.jpg]

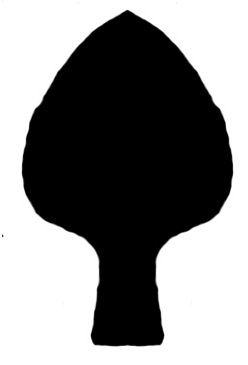

Supplement: Supplementary file 3 — Supplementary Information 3. [file 41598_2022_7823_MOESM3_ESM.zip › Amb_davisi.jpg]

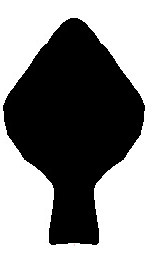

Supplement: Supplementary file 3 — Supplementary Information 3. [file 41598_2022_7823_MOESM3_ESM.zip › Amb_montielae.jpg]

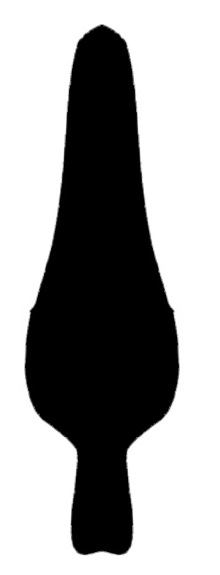

Supplement: Supplementary file 3 — Supplementary Information 3. [file 41598_2022_7823_MOESM3_ESM.zip › Ane_harteni.jpg]

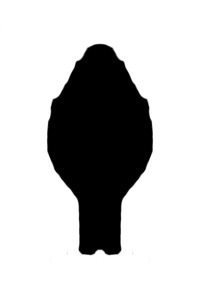

Supplement: Supplementary file 3 — Supplementary Information 3. [file 41598_2022_7823_MOESM3_ESM.zip › Ant_anseli.jpg]

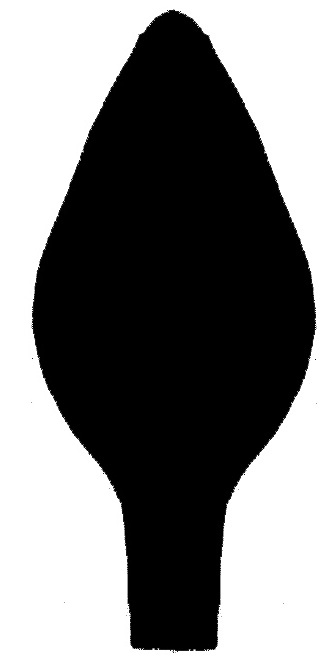

Supplement: Supplementary file 3 — Supplementary Information 3. [file 41598_2022_7823_MOESM3_ESM.zip › Ant_brevipatellatus.jpg]

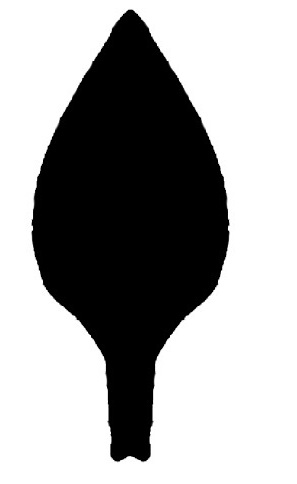

Supplement: Supplementary file 3 — Supplementary Information 3. [file 41598_2022_7823_MOESM3_ESM.zip › Ant_cokendolpheri.jpg]

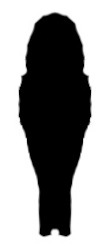

Supplement: Supplementary file 3 — Supplementary Information 3. [file 41598_2022_7823_MOESM3_ESM.zip › Ant_eremita.jpg]

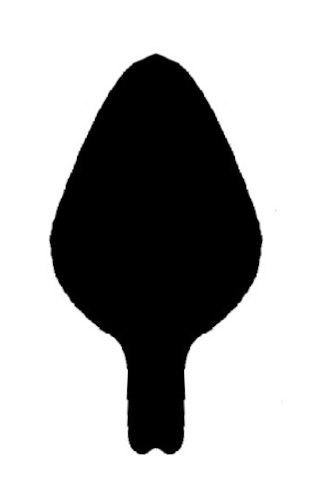

Supplement: Supplementary file 3 — Supplementary Information 3. [file 41598_2022_7823_MOESM3_ESM.zip › Ant_gibarensis.jpg]

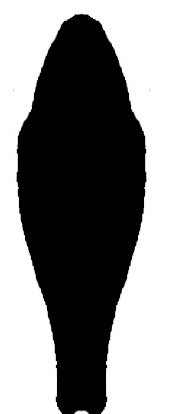

Supplement: Supplementary file 3 — Supplementary Information 3. [file 41598_2022_7823_MOESM3_ESM.zip › Ant_holguin.jpg]

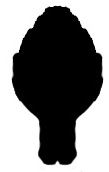

Supplement: Supplementary file 3 — Supplementary Information 3. [file 41598_2022_7823_MOESM3_ESM.zip › Ant_longior.jpg]

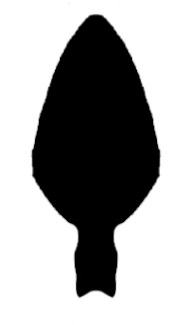

Supplement: Supplementary file 3 — Supplementary Information 3. [file 41598_2022_7823_MOESM3_ESM.zip › Ant_planicauda.jpg]

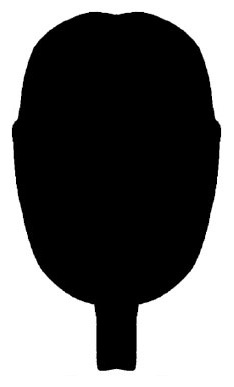

Supplement: Supplementary file 3 — Supplementary Information 3. [file 41598_2022_7823_MOESM3_ESM.zip › Apo_alligator.jpg]

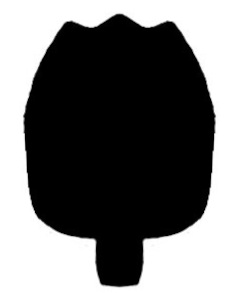

Supplement: Supplementary file 3 — Supplementary Information 3. [file 41598_2022_7823_MOESM3_ESM.zip › Apo_brignolii.jpg]

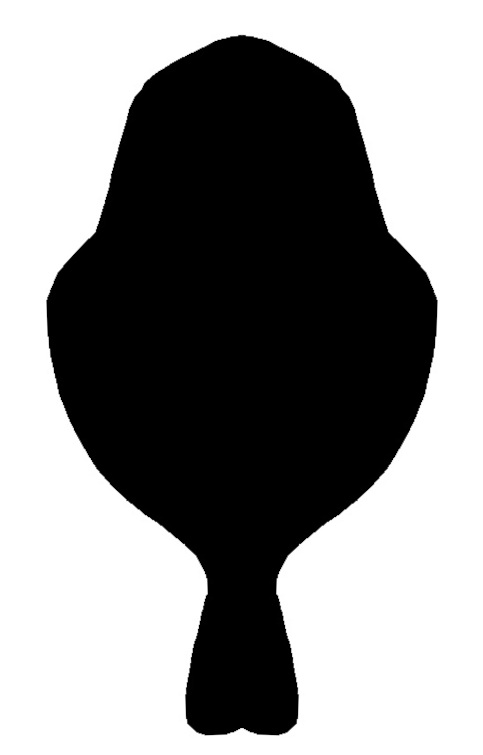

Supplement: Supplementary file 3 — Supplementary Information 3. [file 41598_2022_7823_MOESM3_ESM.zip › Apo_eberhardi.jpg]

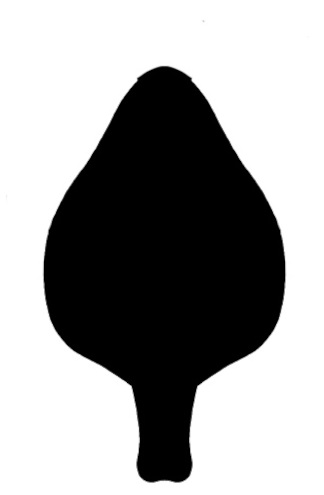

Supplement: Supplementary file 3 — Supplementary Information 3. [file 41598_2022_7823_MOESM3_ESM.zip › Apo_gerlachi.jpg]

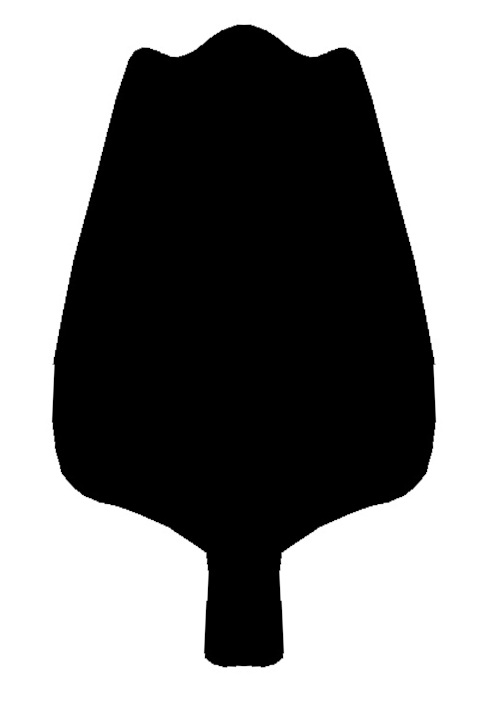

Supplement: Supplementary file 3 — Supplementary Information 3. [file 41598_2022_7823_MOESM3_ESM.zip › Apo_howarthi.jpg]

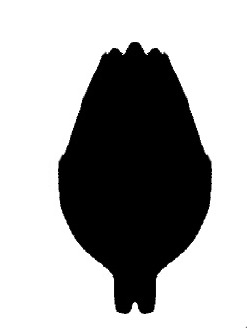

Supplement: Supplementary file 3 — Supplementary Information 3. [file 41598_2022_7823_MOESM3_ESM.zip › Apo_termitarium.jpg]

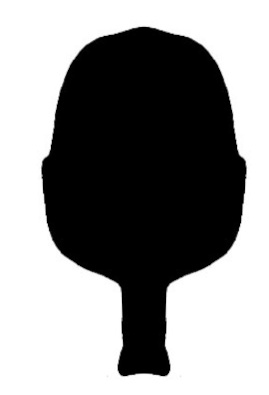

Supplement: Supplementary file 3 — Supplementary Information 3. [file 41598_2022_7823_MOESM3_ESM.zip › Apo_watsoni.jpg]

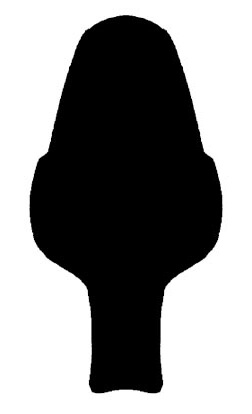

Supplement: Supplementary file 3 — Supplementary Information 3. [file 41598_2022_7823_MOESM3_ESM.zip › Apo_yirrkala.jpg]

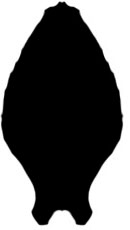

Supplement: Supplementary file 3 — Supplementary Information 3. [file 41598_2022_7823_MOESM3_ESM.zip › Art_liberiensis.jpg]

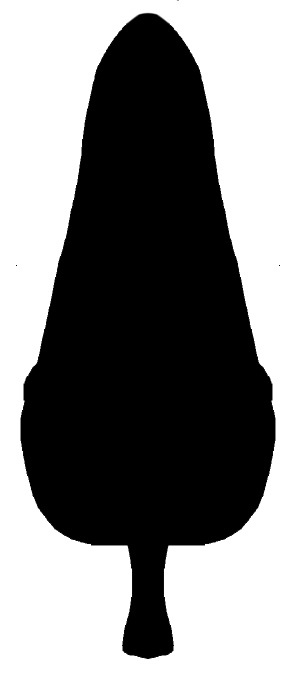

Supplement: Supplementary file 3 — Supplementary Information 3. [file 41598_2022_7823_MOESM3_ESM.zip › Att_baroalba.jpg]

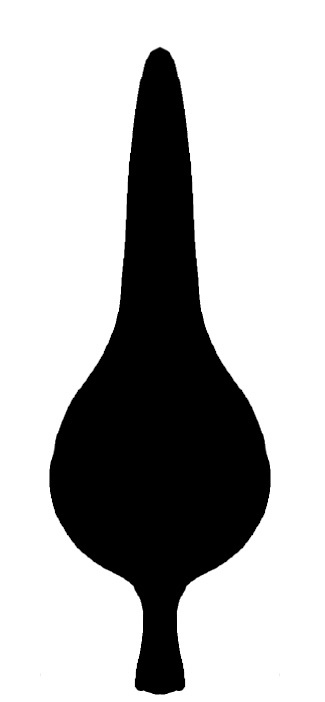

Supplement: Supplementary file 3 — Supplementary Information 3. [file 41598_2022_7823_MOESM3_ESM.zip › Att_cuttacutta.jpg]

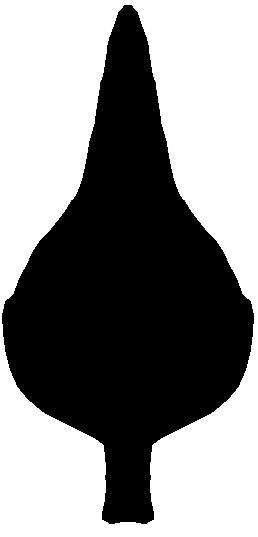

Supplement: Supplementary file 3 — Supplementary Information 3. [file 41598_2022_7823_MOESM3_ESM.zip › Att_mainae.jpg]

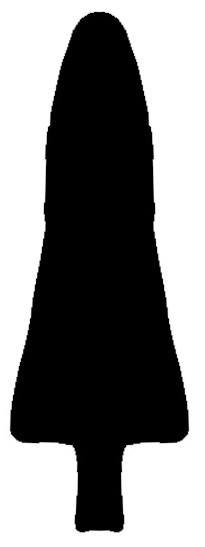

Supplement: Supplementary file 3 — Supplementary Information 3. [file 41598_2022_7823_MOESM3_ESM.zip › Att_radon.jpg]

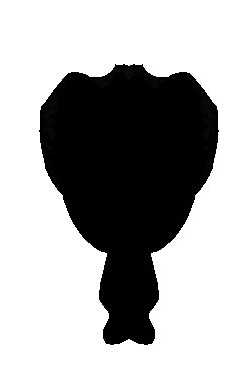

Supplement: Supplementary file 3 — Supplementary Information 3. [file 41598_2022_7823_MOESM3_ESM.zip › Baa_firstmani.jpg]

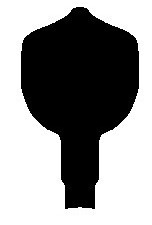

Supplement: Supplementary file 3 — Supplementary Information 3. [file 41598_2022_7823_MOESM3_ESM.zip › Baa_magico.jpg]

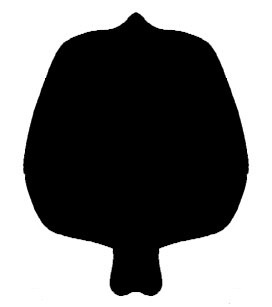

Supplement: Supplementary file 3 — Supplementary Information 3. [file 41598_2022_7823_MOESM3_ESM.zip › Bam_bamaga.jpg]

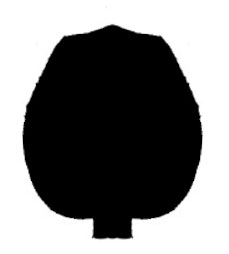

Supplement: Supplementary file 3 — Supplementary Information 3. [file 41598_2022_7823_MOESM3_ESM.zip › Bam_madagassus.jpg]

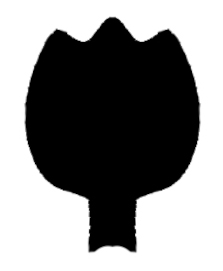

Supplement: Supplementary file 3 — Supplementary Information 3. [file 41598_2022_7823_MOESM3_ESM.zip › Bam_milloti.jpg]

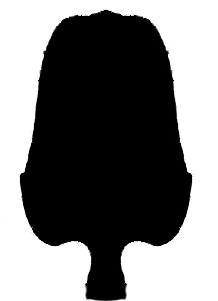

Supplement: Supplementary file 3 — Supplementary Information 3. [file 41598_2022_7823_MOESM3_ESM.zip › Bam_pileti.jpg]

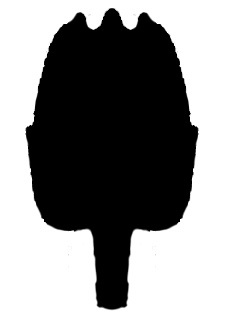

Supplement: Supplementary file 3 — Supplementary Information 3. [file 41598_2022_7823_MOESM3_ESM.zip › Bam_siamensis.jpg]

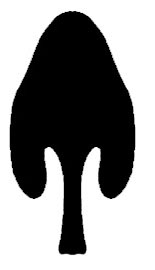

Supplement: Supplementary file 3 — Supplementary Information 3. [file 41598_2022_7823_MOESM3_ESM.zip › Bam_subsolanus.jpg]

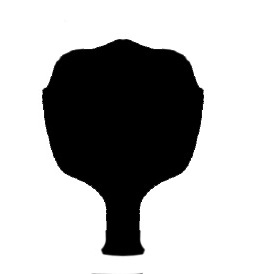

Supplement: Supplementary file 3 — Supplementary Information 3. [file 41598_2022_7823_MOESM3_ESM.zip › Bam_vadoni.jpg]

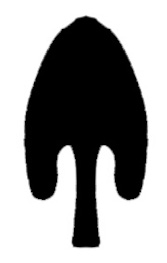

Supplement: Supplementary file 3 — Supplementary Information 3. [file 41598_2022_7823_MOESM3_ESM.zip › Bam_vespertinus.jpg]

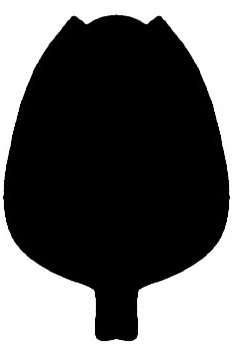

Supplement: Supplementary file 3 — Supplementary Information 3. [file 41598_2022_7823_MOESM3_ESM.zip › Bam_weipa.jpg]

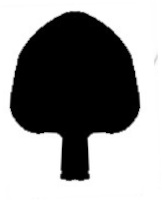

Supplement: Supplementary file 3 — Supplementary Information 3. [file 41598_2022_7823_MOESM3_ESM.zip › Bel_pentalatus.jpg]

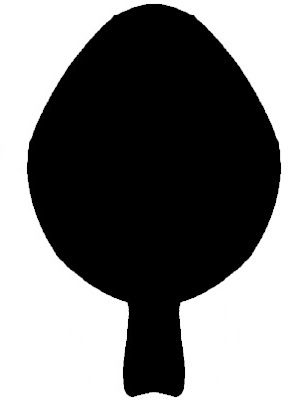

Supplement: Supplementary file 3 — Supplementary Information 3. [file 41598_2022_7823_MOESM3_ESM.zip › Bri_nob.jpg]

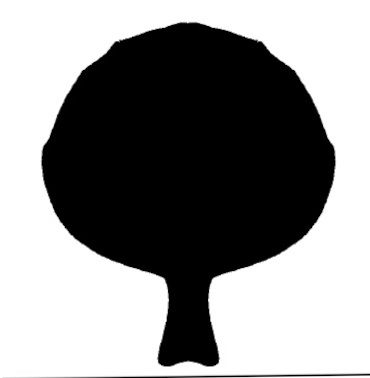

Supplement: Supplementary file 3 — Supplementary Information 3. [file 41598_2022_7823_MOESM3_ESM.zip › Bri_walteri.jpg]

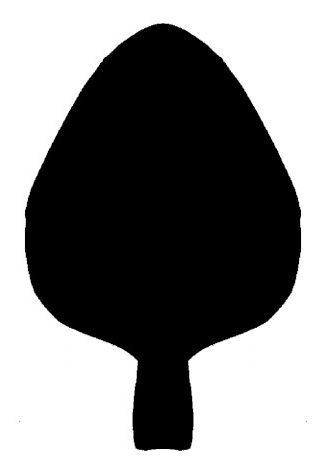

Supplement: Supplementary file 3 — Supplementary Information 3. [file 41598_2022_7823_MOESM3_ESM.zip › Bri_woodwardi.jpg]

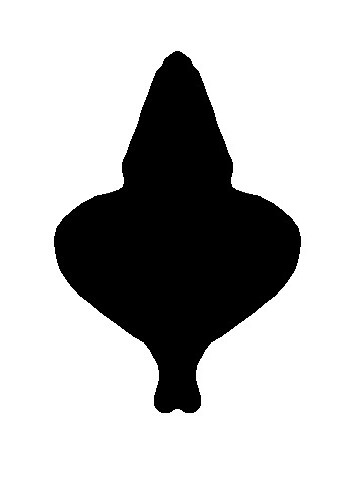

Supplement: Supplementary file 3 — Supplementary Information 3. [file 41598_2022_7823_MOESM3_ESM.zip › Buc_hortuspalmarum.jpg]

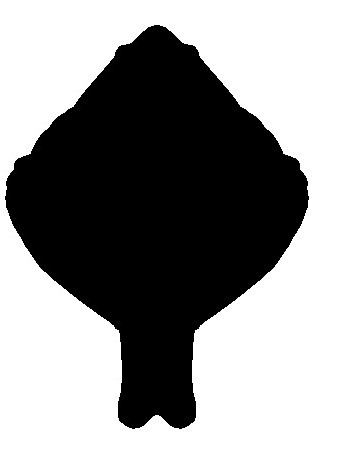

Supplement: Supplementary file 3 — Supplementary Information 3. [file 41598_2022_7823_MOESM3_ESM.zip › Cal_bremensis.jpg]

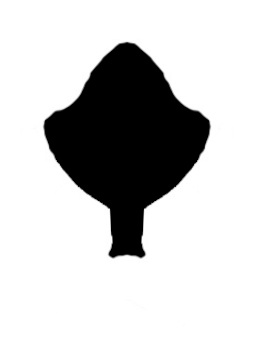

Supplement: Supplementary file 3 — Supplementary Information 3. [file 41598_2022_7823_MOESM3_ESM.zip › Cal_embera.jpg]

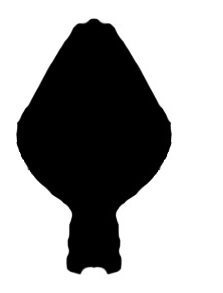

Supplement: Supplementary file 3 — Supplementary Information 3. [file 41598_2022_7823_MOESM3_ESM.zip › Cal_nutabe.jpg]

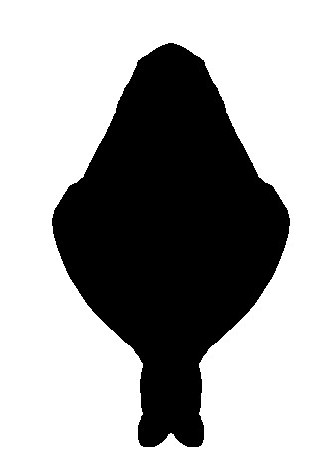

Supplement: Supplementary file 3 — Supplementary Information 3. [file 41598_2022_7823_MOESM3_ESM.zip › Cal_valenciorum.jpg]

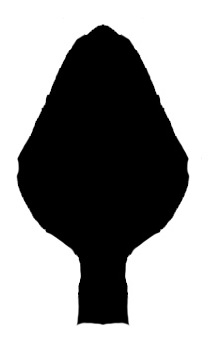

Supplement: Supplementary file 3 — Supplementary Information 3. [file 41598_2022_7823_MOESM3_ESM.zip › Can_xikrin.jpg]

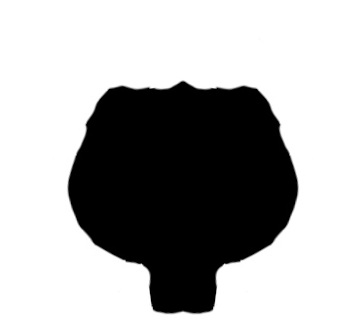

Supplement: Supplementary file 3 — Supplementary Information 3. [file 41598_2022_7823_MOESM3_ESM.zip › Car_laurae.jpg]

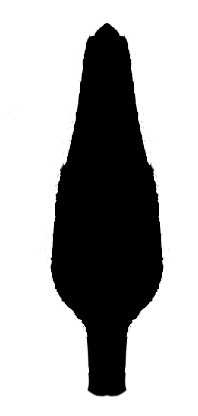

Supplement: Supplementary file 3 — Supplementary Information 3. [file 41598_2022_7823_MOESM3_ESM.zip › Cla_claviger.jpg]

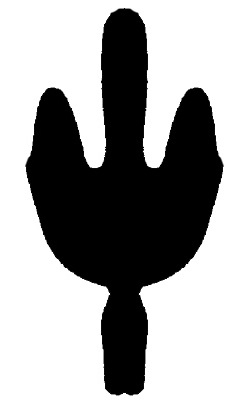

Supplement: Supplementary file 3 — Supplementary Information 3. [file 41598_2022_7823_MOESM3_ESM.zip › Cok_ramosiArmas.jpg]

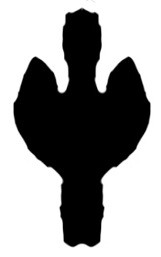

Supplement: Supplementary file 3 — Supplementary Information 3. [file 41598_2022_7823_MOESM3_ESM.zip › Cok_ramosiTer.jpg]

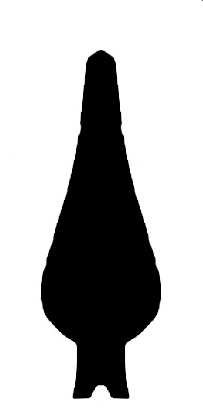

Supplement: Supplementary file 3 — Supplementary Information 3. [file 41598_2022_7823_MOESM3_ESM.zip › Col_quindio.jpg]

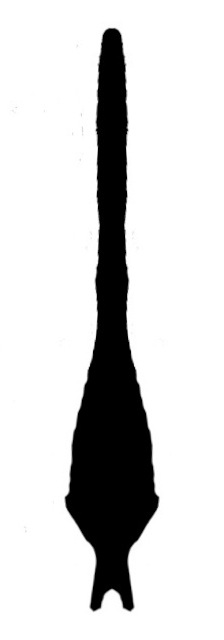

Supplement: Supplementary file 3 — Supplementary Information 3. [file 41598_2022_7823_MOESM3_ESM.zip › Col_truncatus.jpg]

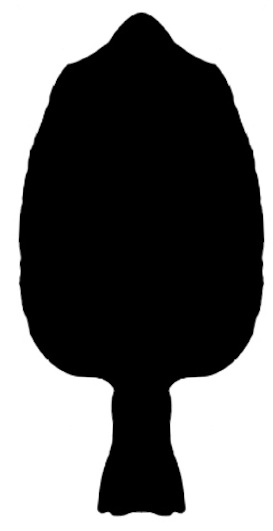

Supplement: Supplementary file 3 — Supplementary Information 3. [file 41598_2022_7823_MOESM3_ESM.zip › Cut_rowlandiArmas.jpg]

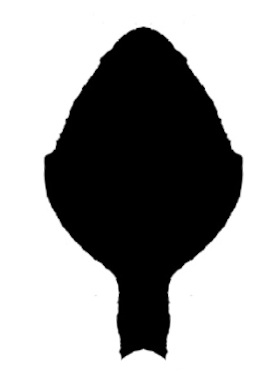

Supplement: Supplementary file 3 — Supplementary Information 3. [file 41598_2022_7823_MOESM3_ESM.zip › Cut_rowlandiDumi.jpg]

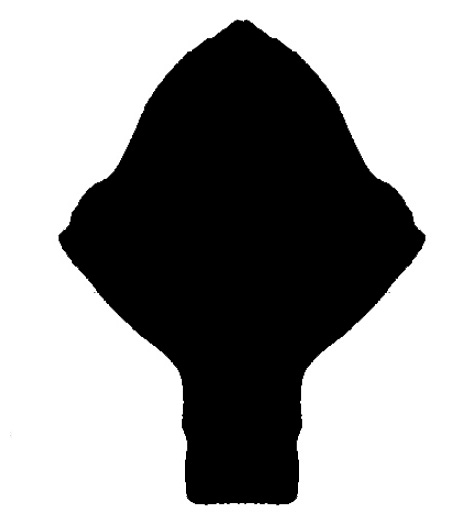

Supplement: Supplementary file 3 — Supplementary Information 3. [file 41598_2022_7823_MOESM3_ESM.zip › Cuz_armasi.jpg]

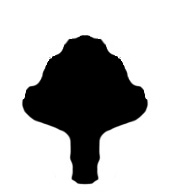

Supplement: Supplementary file 3 — Supplementary Information 3. [file 41598_2022_7823_MOESM3_ESM.zip › Cuz_montanusTer17.jpg]

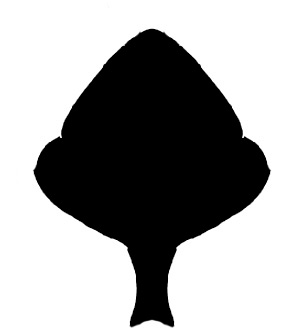

Supplement: Supplementary file 3 — Supplementary Information 3. [file 41598_2022_7823_MOESM3_ESM.zip › Cuz_montanusTer4.jpg]

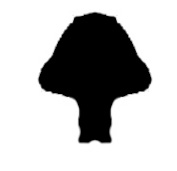

Supplement: Supplementary file 3 — Supplementary Information 3. [file 41598_2022_7823_MOESM3_ESM.zip › Cuz_sheylae.jpg]

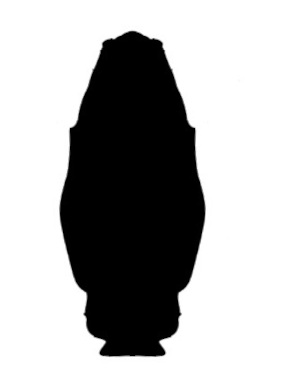

Supplement: Supplementary file 3 — Supplementary Information 3. [file 41598_2022_7823_MOESM3_ESM.zip › Dra_anachoretus.jpg]

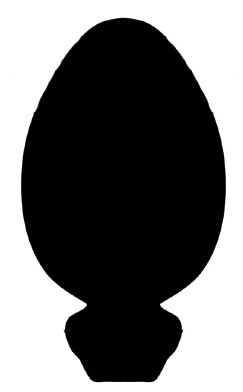

Supplement: Supplementary file 3 — Supplementary Information 3. [file 41598_2022_7823_MOESM3_ESM.zip › Dra_belalugosii.jpg]

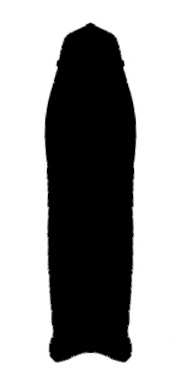

Supplement: Supplementary file 3 — Supplementary Information 3. [file 41598_2022_7823_MOESM3_ESM.zip › Dra_bramstokeri.jpg]

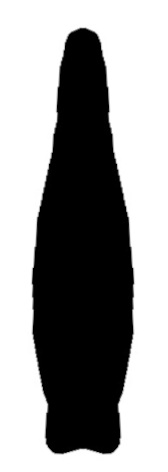

Supplement: Supplementary file 3 — Supplementary Information 3. [file 41598_2022_7823_MOESM3_ESM.zip › Dra_brooksi.jpg]

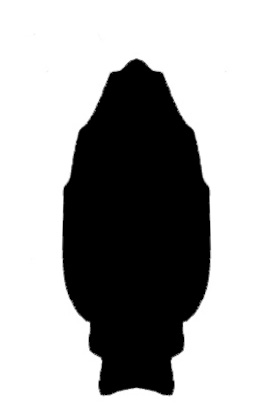

Supplement: Supplementary file 3 — Supplementary Information 3. [file 41598_2022_7823_MOESM3_ESM.zip › Dra_bythius.jpg]

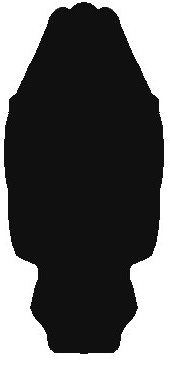

Supplement: Supplementary file 3 — Supplementary Information 3. [file 41598_2022_7823_MOESM3_ESM.zip › Dra_christopherleei.JPG]

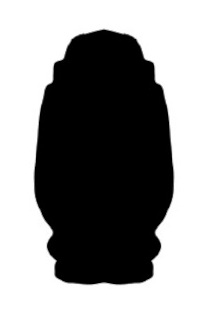

Supplement: Supplementary file 3 — Supplementary Information 3. [file 41598_2022_7823_MOESM3_ESM.zip › Dra_eremius.jpg]

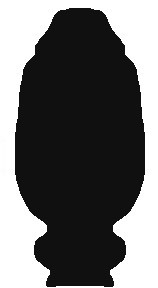

Supplement: Supplementary file 3 — Supplementary Information 3. [file 41598_2022_7823_MOESM3_ESM.zip › Dra_gnophicola.JPG]

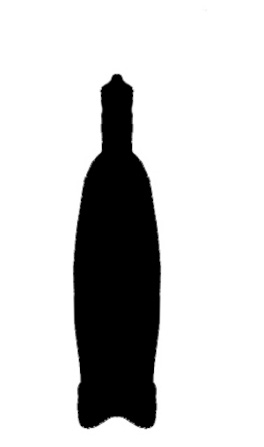

Supplement: Supplementary file 3 — Supplementary Information 3. [file 41598_2022_7823_MOESM3_ESM.zip › Dra_julianneae.jpg]

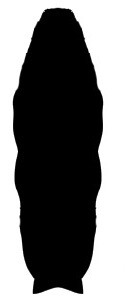

Supplement: Supplementary file 3 — Supplementary Information 3. [file 41598_2022_7823_MOESM3_ESM.zip › Dra_karenbassettae.jpg]

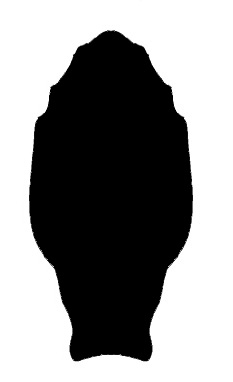

Supplement: Supplementary file 3 — Supplementary Information 3. [file 41598_2022_7823_MOESM3_ESM.zip › Dra_kryptus.jpg]

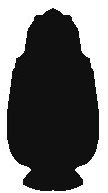

Supplement: Supplementary file 3 — Supplementary Information 3. [file 41598_2022_7823_MOESM3_ESM.zip › Dra_mckechnieorum.JPG]

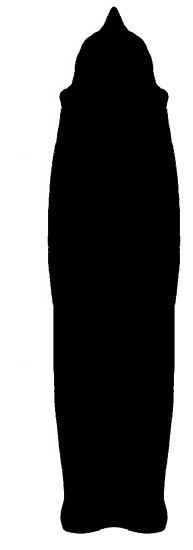

Supplement: Supplementary file 3 — Supplementary Information 3. [file 41598_2022_7823_MOESM3_ESM.zip › Dra_neoanthropus.jpg]

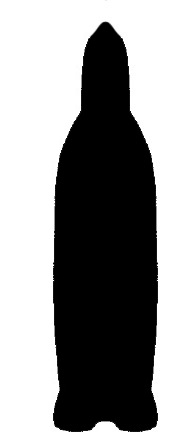

Supplement: Supplementary file 3 — Supplementary Information 3. [file 41598_2022_7823_MOESM3_ESM.zip › Dra_vinei.jpg]

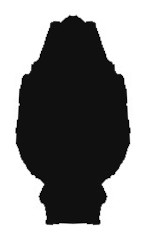

Supplement: Supplementary file 3 — Supplementary Information 3. [file 41598_2022_7823_MOESM3_ESM.zip › Dra_warramboo.JPG]

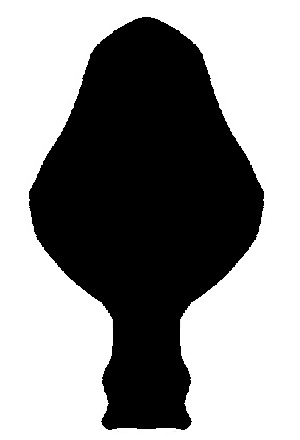

Supplement: Supplementary file 3 — Supplementary Information 3. [file 41598_2022_7823_MOESM3_ESM.zip › Dum_decuiRR.jpg]

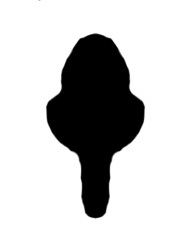

Supplement: Supplementary file 3 — Supplementary Information 3. [file 41598_2022_7823_MOESM3_ESM.zip › Dum_decuiTer.jpg]

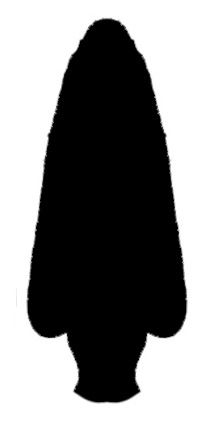

Supplement: Supplementary file 3 — Supplementary Information 3. [file 41598_2022_7823_MOESM3_ESM.zip › Eni_eruptoclausus.jpg]

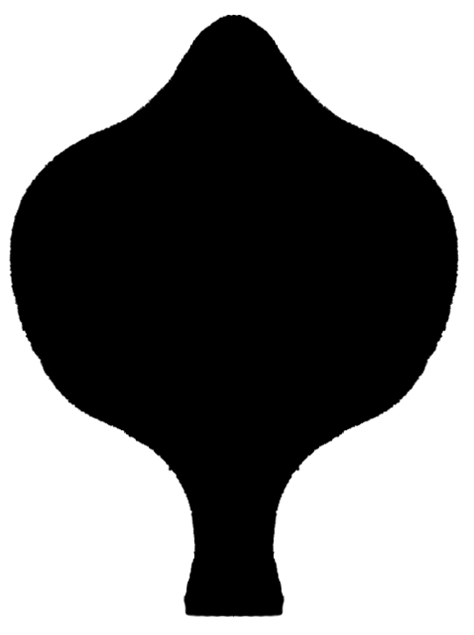

Supplement: Supplementary file 3 — Supplementary Information 3. [file 41598_2022_7823_MOESM3_ESM.zip › Gen_sp1.jpg]

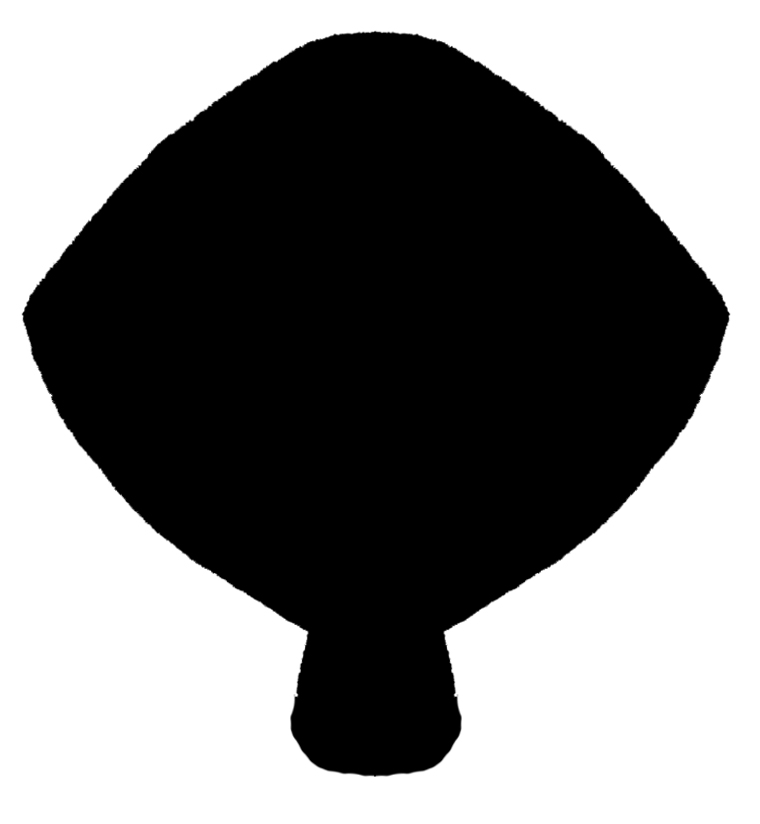

Supplement: Supplementary file 3 — Supplementary Information 3. [file 41598_2022_7823_MOESM3_ESM.zip › Gen_sp2.jpg]

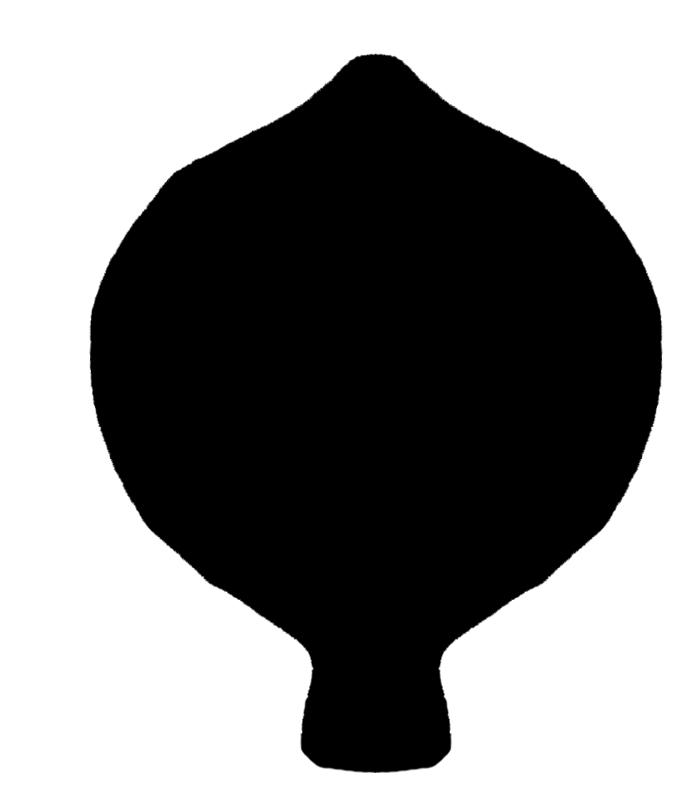

Supplement: Supplementary file 3 — Supplementary Information 3. [file 41598_2022_7823_MOESM3_ESM.zip › Gen_sp3.jpg]

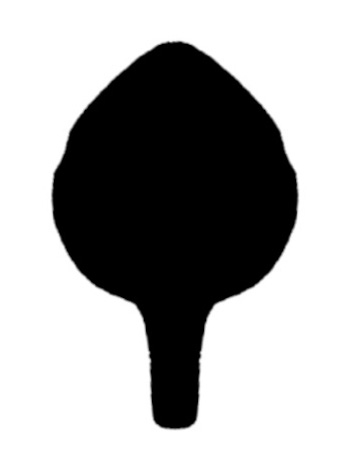

Supplement: Supplementary file 3 — Supplementary Information 3. [file 41598_2022_7823_MOESM3_ESM.zip › Gua_armatus.jpg]

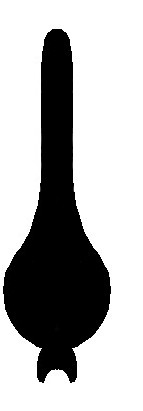

Supplement: Supplementary file 3 — Supplementary Information 3. [file 41598_2022_7823_MOESM3_ESM.zip › Han_acrocaudatus.jpg]

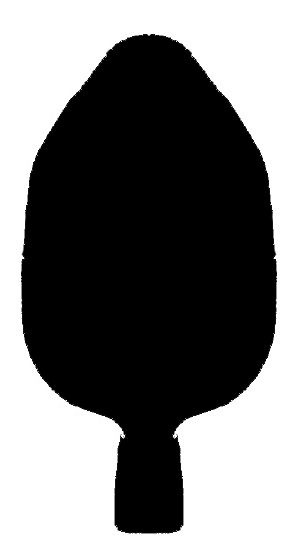

Supplement: Supplementary file 3 — Supplementary Information 3. [file 41598_2022_7823_MOESM3_ESM.zip › Han_centralis.jpg]

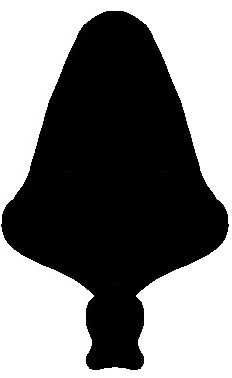

Supplement: Supplementary file 3 — Supplementary Information 3. [file 41598_2022_7823_MOESM3_ESM.zip › Han_drakos.jpg]

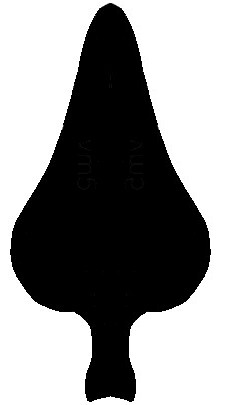

Supplement: Supplementary file 3 — Supplementary Information 3. [file 41598_2022_7823_MOESM3_ESM.zip › Han_gladiator.jpg]

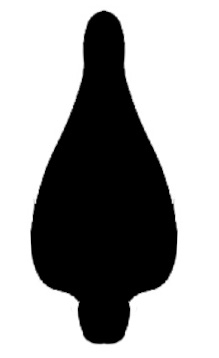

Supplement: Supplementary file 3 — Supplementary Information 3. [file 41598_2022_7823_MOESM3_ESM.zip › Han_humbertoi.jpg]

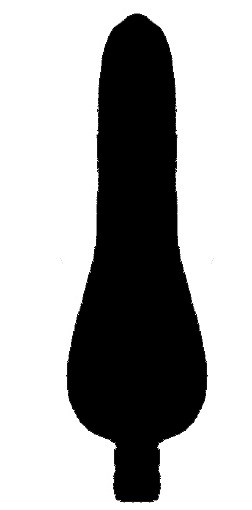

Supplement: Supplementary file 3 — Supplementary Information 3. [file 41598_2022_7823_MOESM3_ESM.zip › Han_mumai1.jpg]

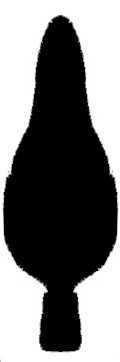

Supplement: Supplementary file 3 — Supplementary Information 3. [file 41598_2022_7823_MOESM3_ESM.zip › Han_mumai2.jpg]

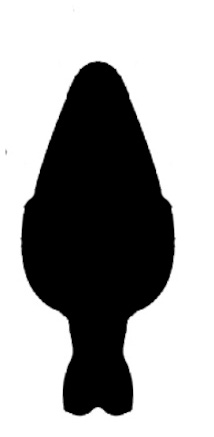

Supplement: Supplementary file 3 — Supplementary Information 3. [file 41598_2022_7823_MOESM3_ESM.zip › Han_selva.jpg]

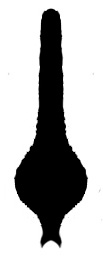

Supplement: Supplementary file 3 — Supplementary Information 3. [file 41598_2022_7823_MOESM3_ESM.zip › Han_simonisHS05.jpg]

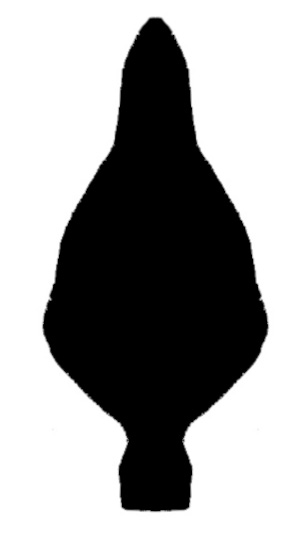

Supplement: Supplementary file 3 — Supplementary Information 3. [file 41598_2022_7823_MOESM3_ESM.zip › Han_simonisRR79.jpg]

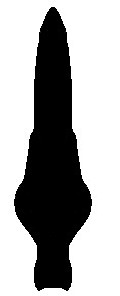

Supplement: Supplementary file 3 — Supplementary Information 3. [file 41598_2022_7823_MOESM3_ESM.zip › Han_surinamensis.jpg]

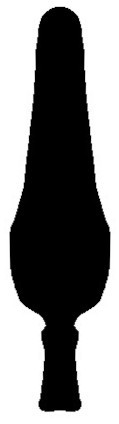

Supplement: Supplementary file 3 — Supplementary Information 3. [file 41598_2022_7823_MOESM3_ESM.zip › Han_tobago.jpg]

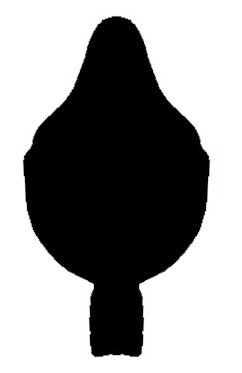

Supplement: Supplementary file 3 — Supplementary Information 3. [file 41598_2022_7823_MOESM3_ESM.zip › Han_trinidanus.jpg]

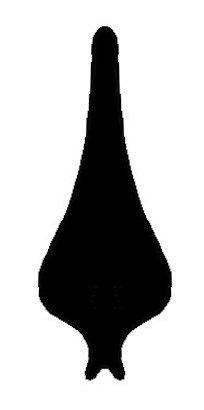

Supplement: Supplementary file 3 — Supplementary Information 3. [file 41598_2022_7823_MOESM3_ESM.zip › Han_urbanii.jpg]
