# Supplementary figures and images for: Patterns in schizomid flagellum shape from elliptical Fourier analysis (part 5 of 5)
Source: Sci Rep. 2022 Mar 10;12:3896. doi: 10.1038/s41598-022-07823-y (PMC8913634; doi:10.1038/s41598-022-07823-y)

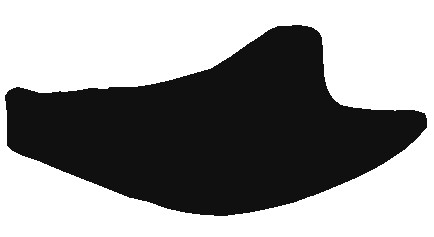

Supplement: Supplementary file 5 — Supplementary Information 5. [file 41598_2022_7823_MOESM5_ESM.zip › Row_longipalpus.jpg]

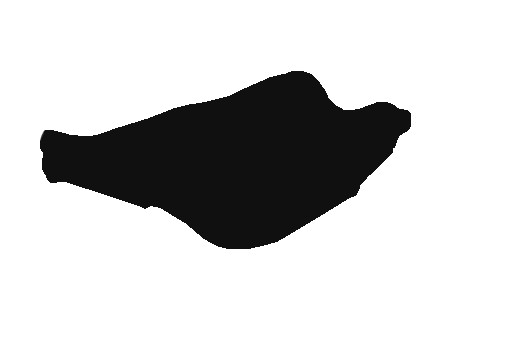

Supplement: Supplementary file 5 — Supplementary Information 5. [file 41598_2022_7823_MOESM5_ESM.zip › Row_marianae.jpg]

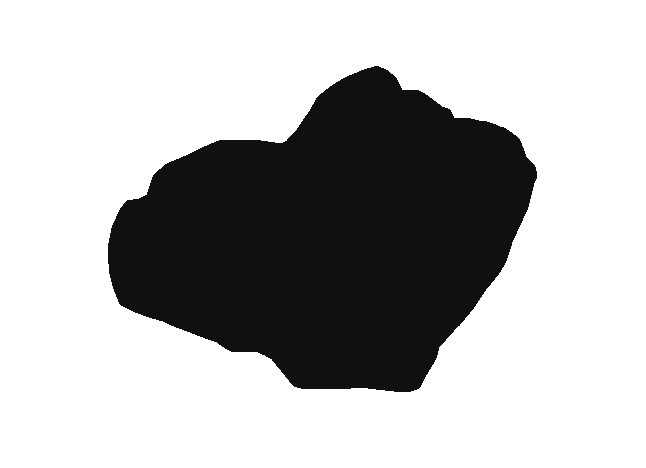

Supplement: Supplementary file 5 — Supplementary Information 5. [file 41598_2022_7823_MOESM5_ESM.zip › Row_martinezi.jpg]

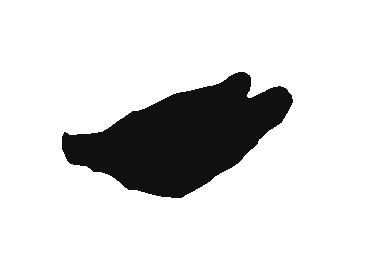

Supplement: Supplementary file 5 — Supplementary Information 5. [file 41598_2022_7823_MOESM5_ESM.zip › Row_melici.jpg]

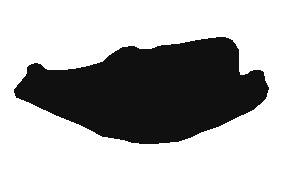

Supplement: Supplementary file 5 — Supplementary Information 5. [file 41598_2022_7823_MOESM5_ESM.zip › Row_mixtus.jpg]

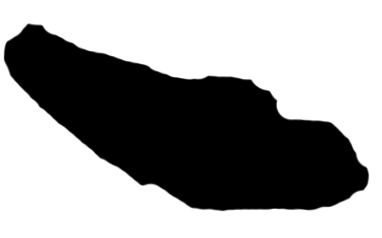

Supplement: Supplementary file 5 — Supplementary Information 5. [file 41598_2022_7823_MOESM5_ESM.zip › Row_moa.jpg]

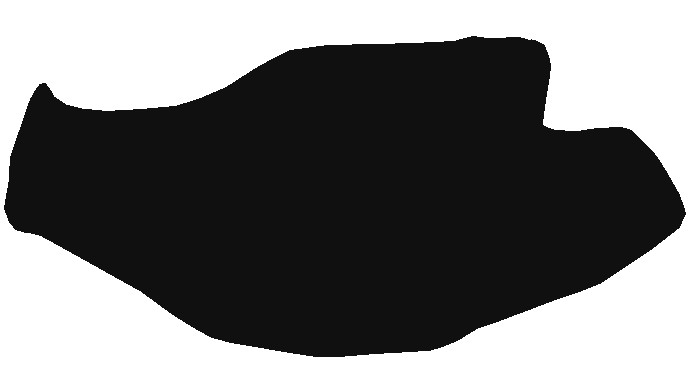

Supplement: Supplementary file 5 — Supplementary Information 5. [file 41598_2022_7823_MOESM5_ESM.zip › Row_monensis.jpg]

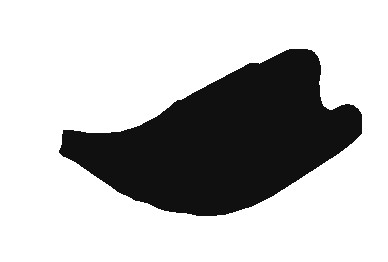

Supplement: Supplementary file 5 — Supplementary Information 5. [file 41598_2022_7823_MOESM5_ESM.zip › Row_monticola.jpg]

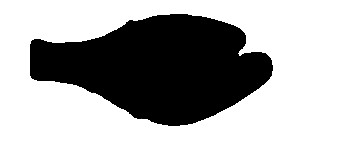

Supplement: Supplementary file 6 — Supplementary Information 6. [file 41598_2022_7823_MOESM6_ESM.zip › Row_naranjo.jpg]

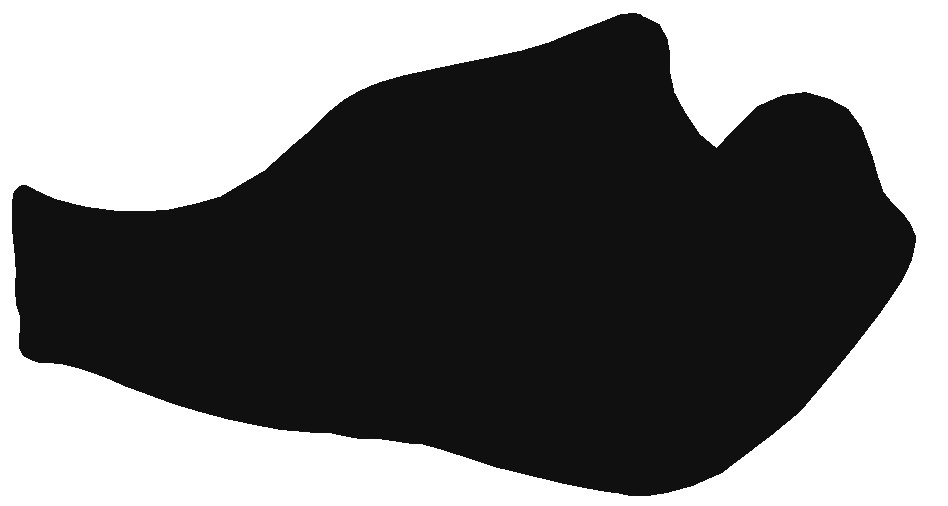

Supplement: Supplementary file 6 — Supplementary Information 6. [file 41598_2022_7823_MOESM6_ESM.zip › Row_peckorum.jpg]

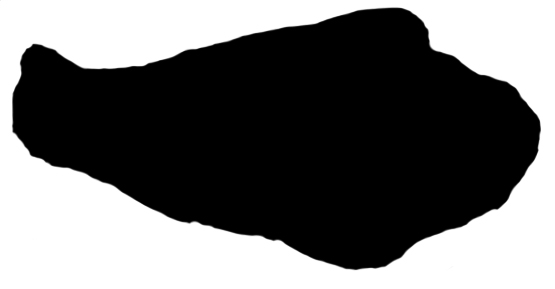

Supplement: Supplementary file 6 — Supplementary Information 6. [file 41598_2022_7823_MOESM6_ESM.zip › Row_pedrosoi.jpg]

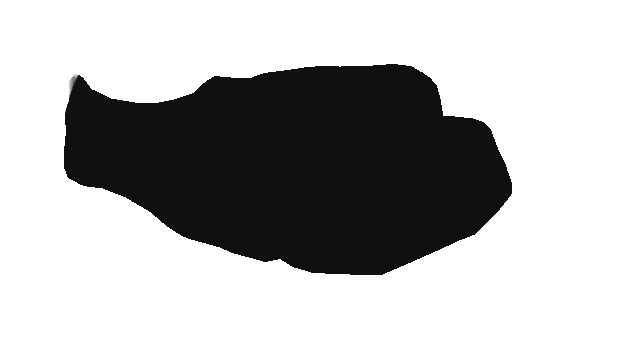

Supplement: Supplementary file 6 — Supplementary Information 6. [file 41598_2022_7823_MOESM6_ESM.zip › Row_potiguar.jpg]

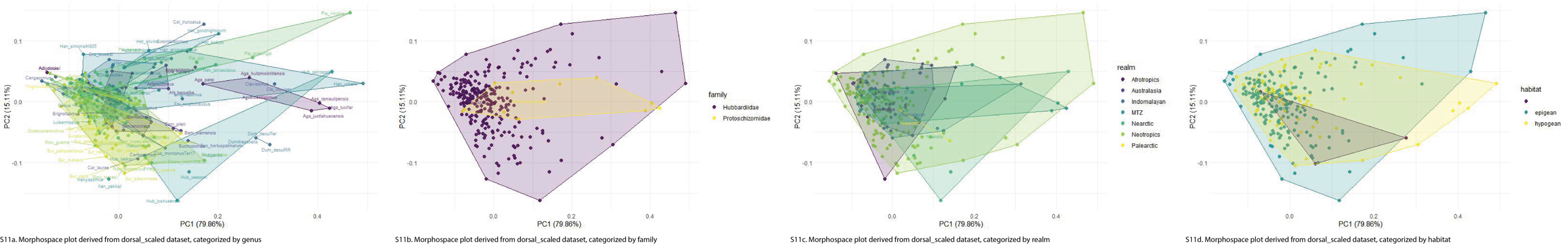

Supplement: Supplementary file 11 — Supplementary Information 11. [file 41598_2022_7823_MOESM11_ESM.png]

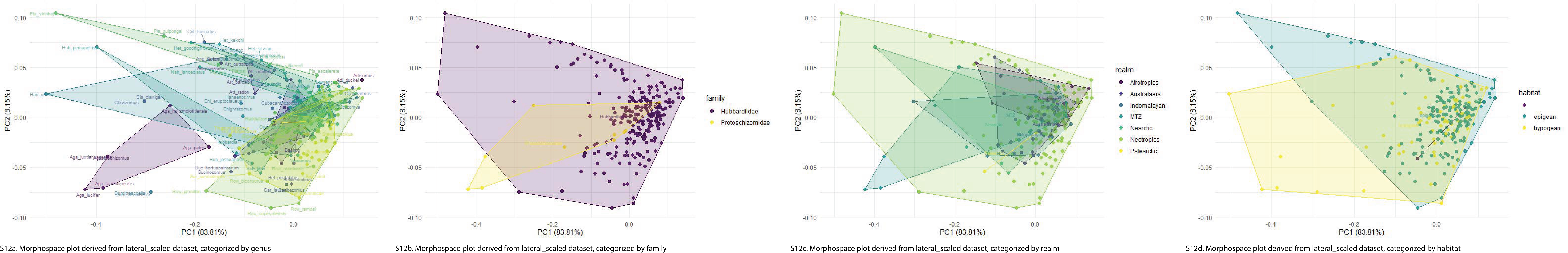

Supplement: Supplementary file 12 — Supplementary Information 12. [file 41598_2022_7823_MOESM12_ESM.png]
