# Supplementary material for: Patterns in schizomid flagellum shape from elliptical Fourier analysis
Source: Sci Rep. 2022 Mar 10;12:3896. doi: 10.1038/s41598-022-07823-y (PMC8913634; doi:10.1038/s41598-022-07823-y)
Supplement: Supplementary file 15 — Supplementary Information 15. [file 41598_2022_7823_MOESM15_ESM.pdf]

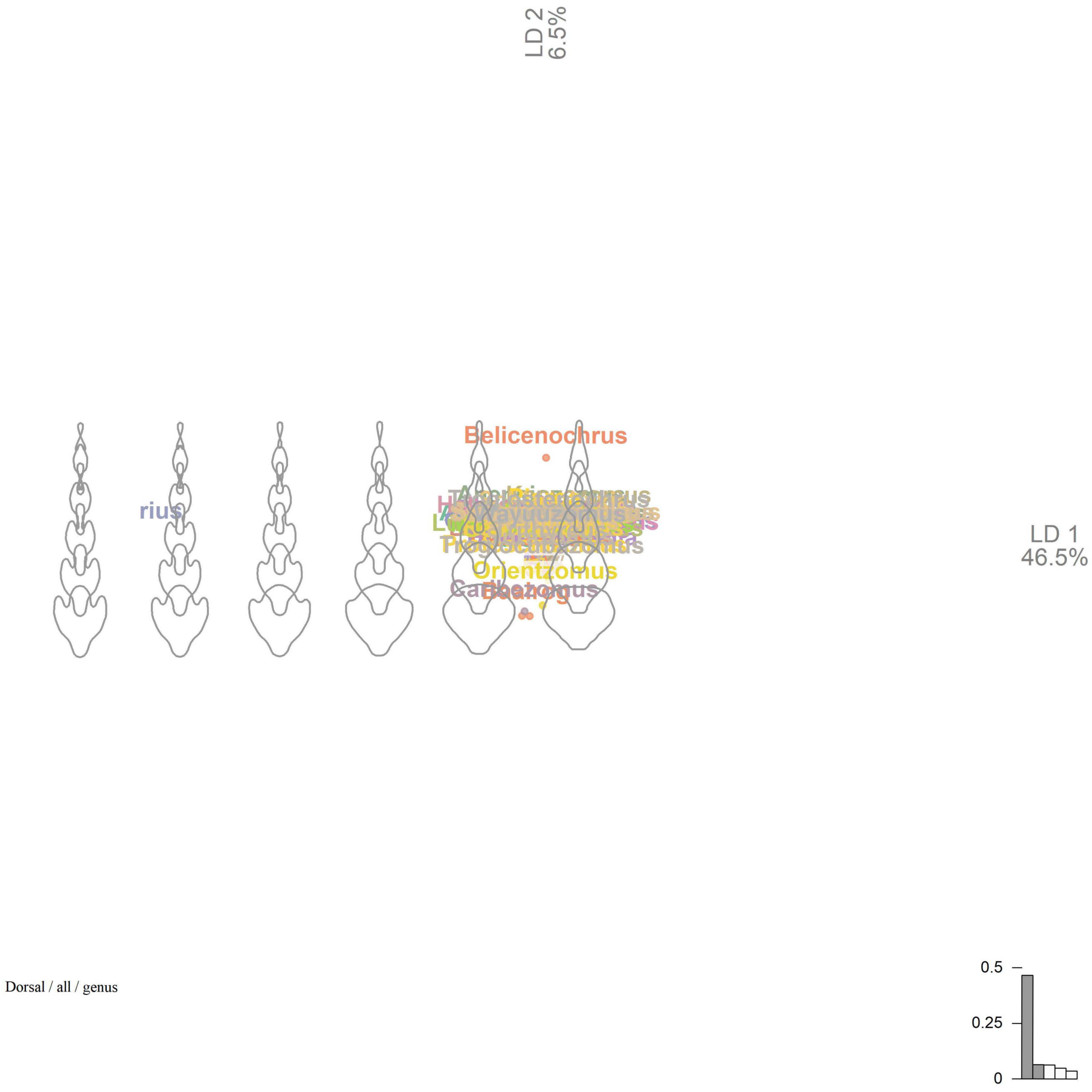
LD 2
6.5%
Belicenochrus
rius
Orientzomus
LD 1
46.5%
Dorsal / all / genus
0.5
0.25
0

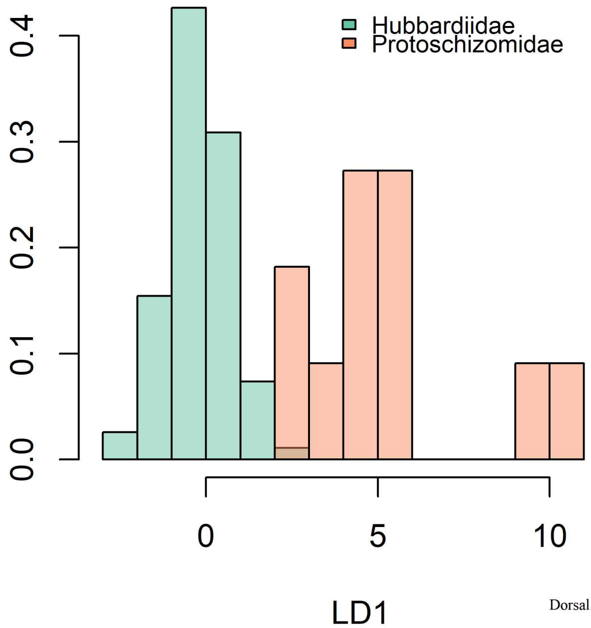

Dorsal / all / family

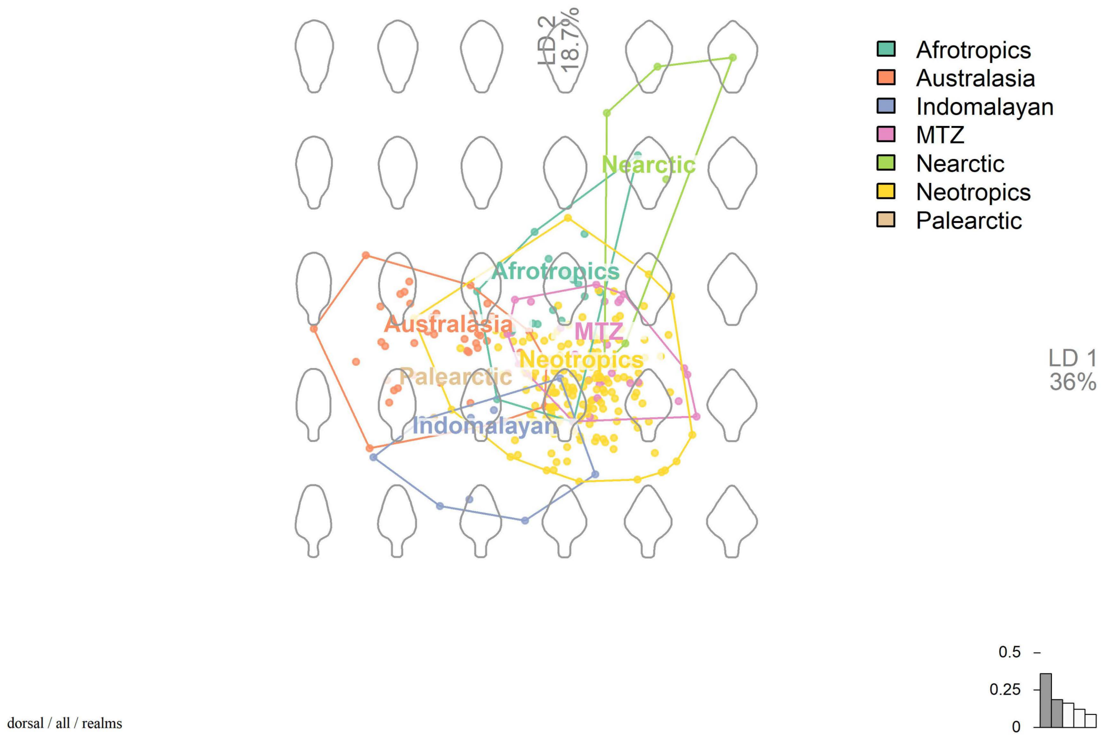

LD 2
18.7%
Afrotropics
Australasia
Indomalayan
MTZ
Nearctic
Neotropics
Palearctic
LD 1
36%
0.5
0.25
0
dorsal / all / realms

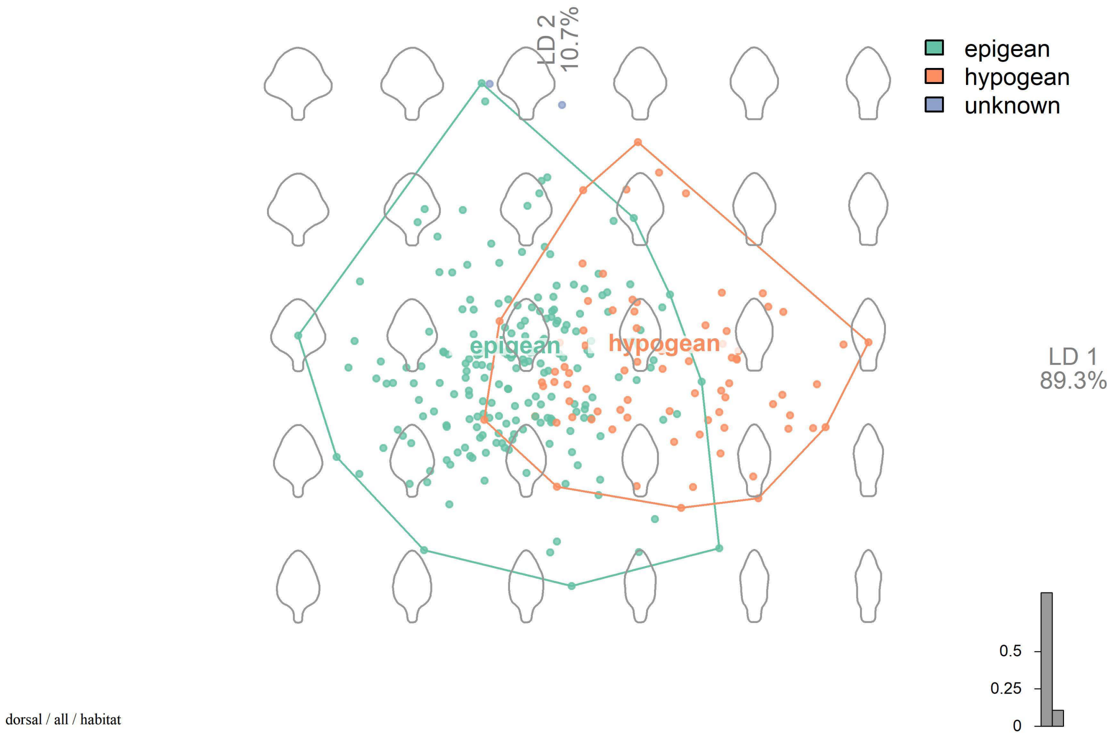
LD 2
10.7%
epigean
hypogean
unknown
epigean
hypogean
LD 1
89.3%
0.5
0.25
0
dorsal / all / habitat

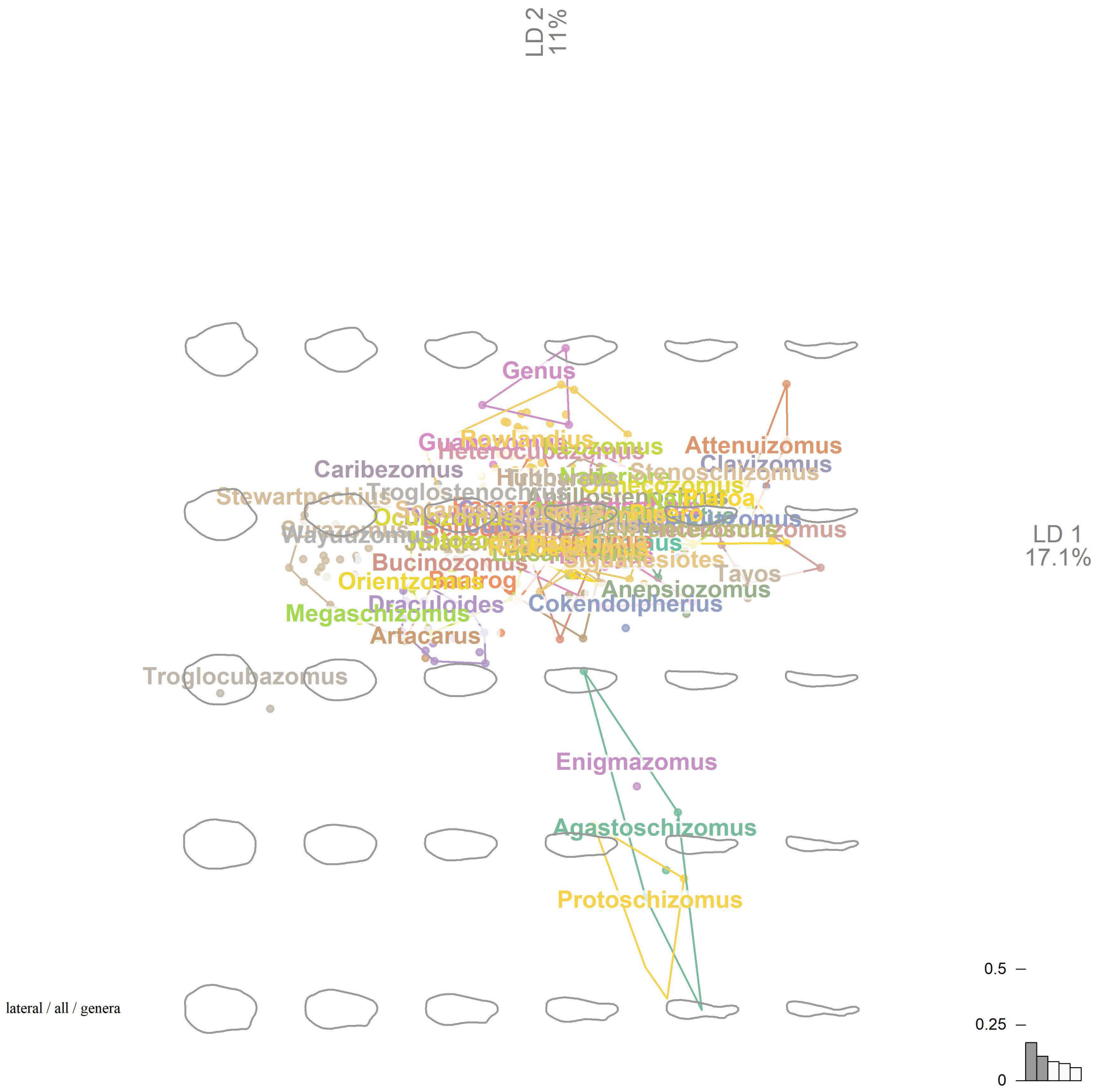

LD 2
11%
LD 1
17.1%
Genus
Attenuizomus
Clavizomus
Caribezomus
Stewartpeckius
Troglostenochrus
Stenoschizomus
Bucinozomus
Orientzomus
Draculoides
Megaschizomus
Artacarus
Cokendolpherius
Anepsiozomus
Tayos
Troglocubazomus
Enigmazomus
Agastoschizomus
Protoschizomus
lateral / all / genera
0.5
0.25
0

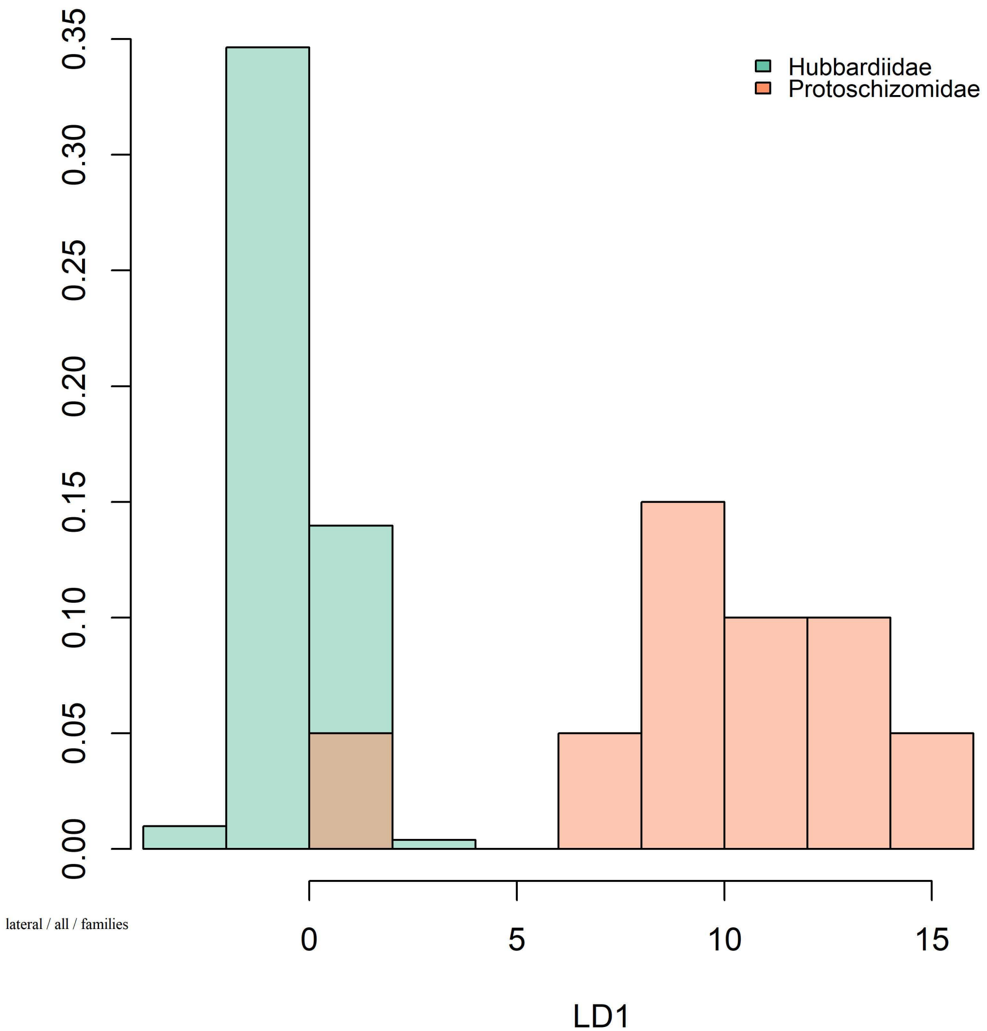
Hubbardiidae
Protoschizomidae
0.00
0.05
0.10
0.15
0.20
0.25
0.30
0.35
0
5
10
15
LD1
lateral / all / families

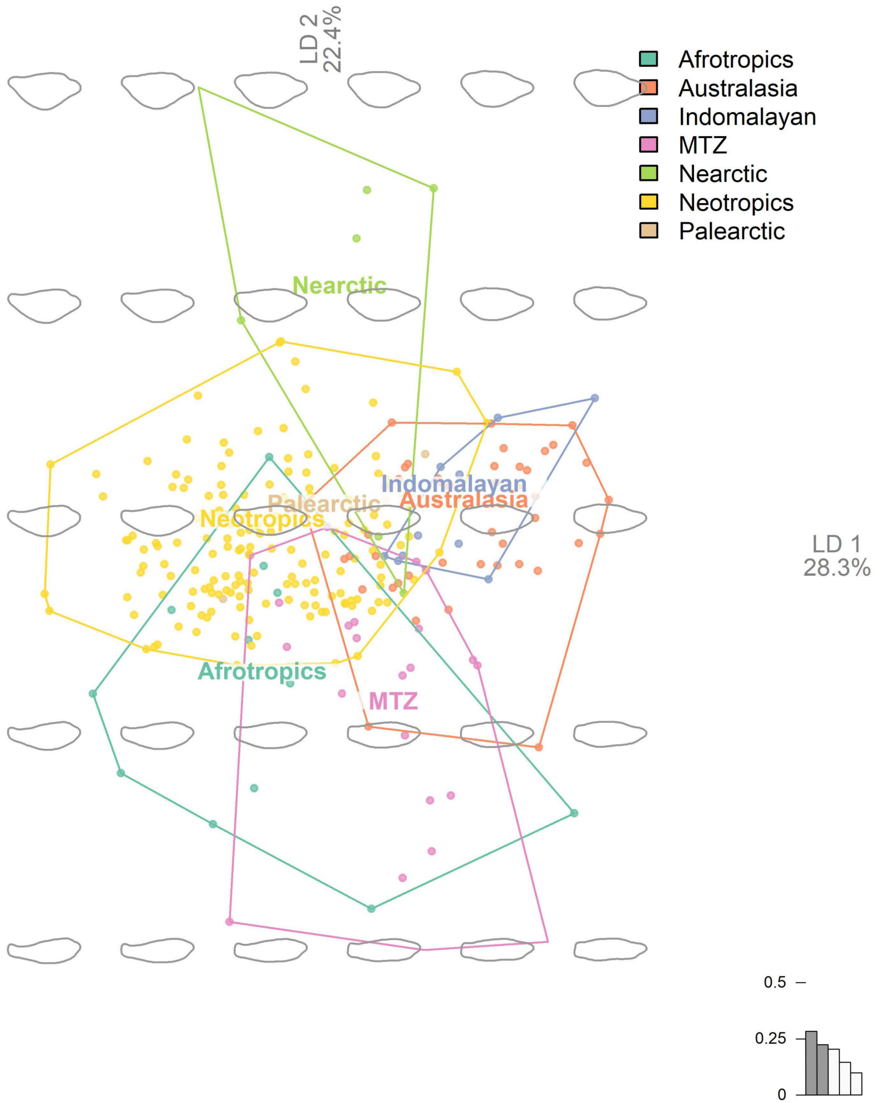

lateral / all/ realm

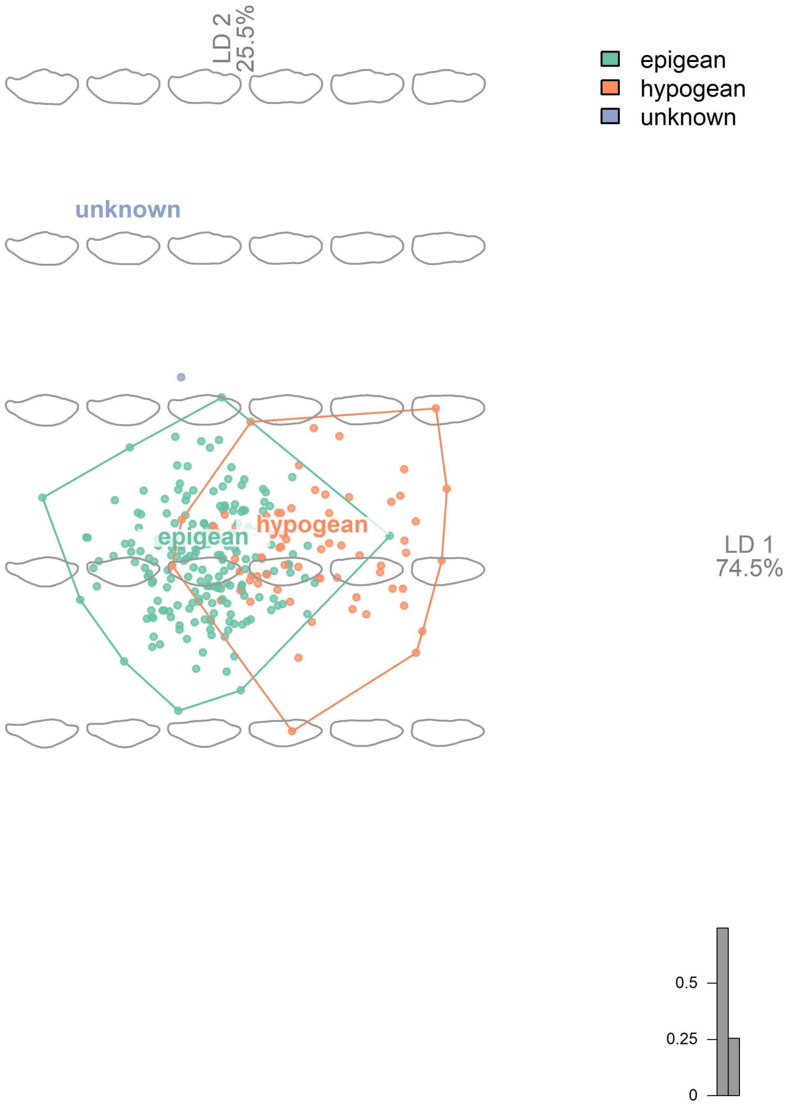

lateral / all / habitat

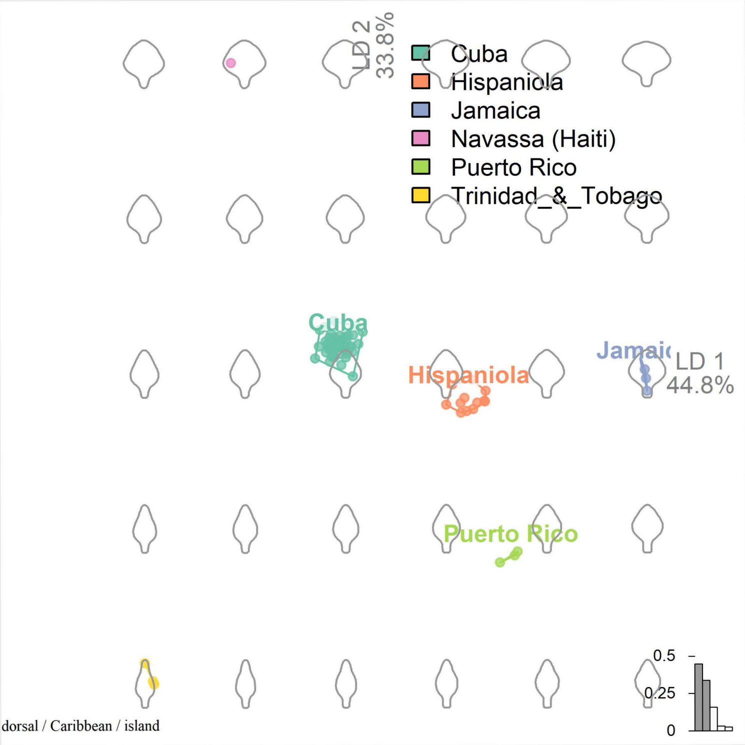
LD 2
33.8%
Cuba
Hispaniola
Jamaica
Navassa (Haiti)
Puerto Rico
Trinidad_&_Tobago
Cuba
Jamaic
LD 1
44.8%
Hispaniola
Puerto Rico
0.5
0.25
0
dorsal / Caribbean / island

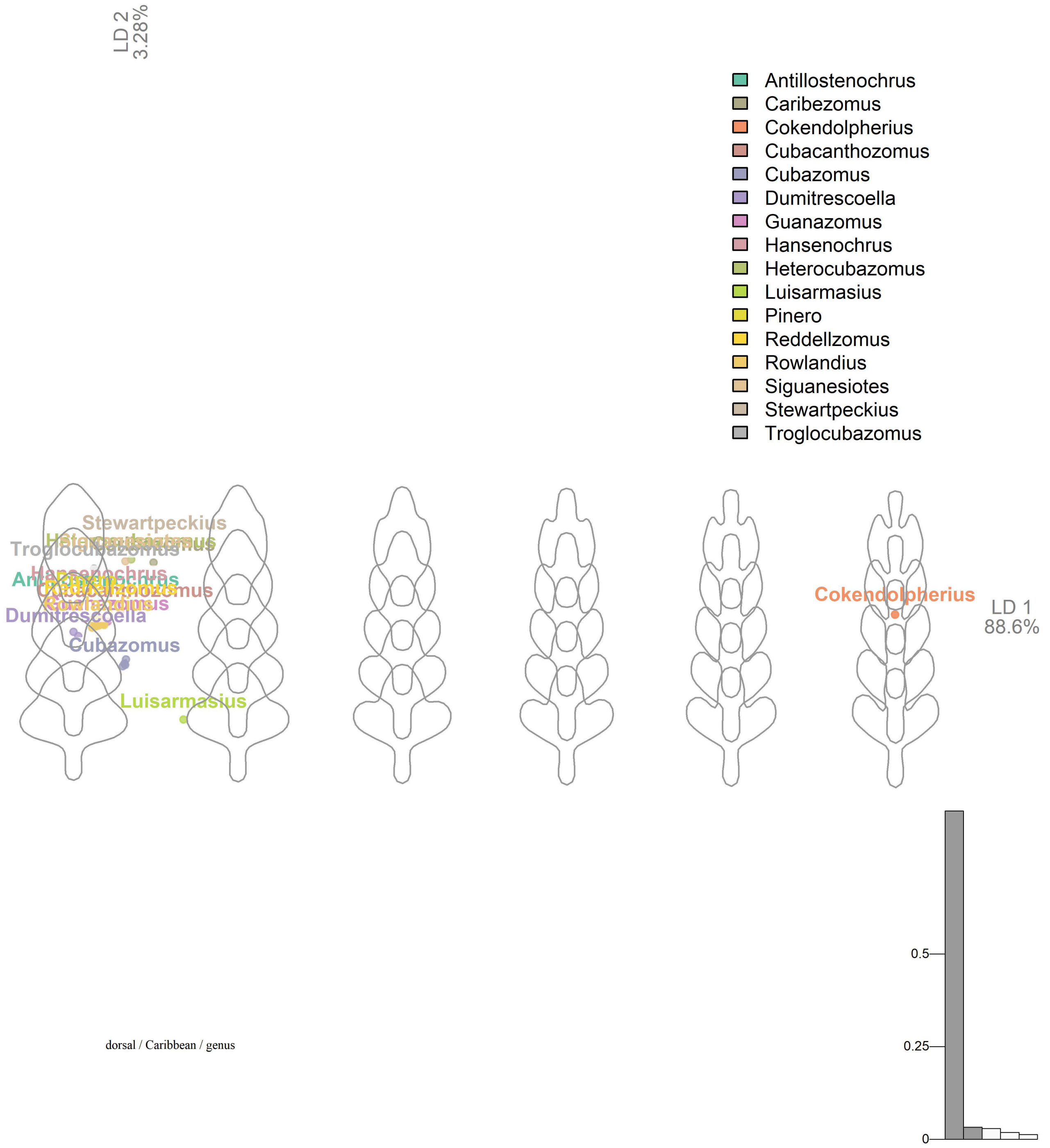
LD 2
3.28%
Antillostenochrus
Caribezomus
Cokendolpherius
Cubacanthozomus
Cubazomus
Dumitrescoella
Guanazomus
Hansenochrus
Heterocubazomus
Luisarmasius
Pinero
Reddellzomus
Rowlandius
Siguanesiotes
Stewartpeckius
Troglocubazomus
Stewartpeckius
Troglocubazomus
Dumitrescoella
Cubazomus
Luisarmasius
Cokendolpherius
LD 1
88.6%
0.5
0.25
0
dorsal / Caribbean / genus

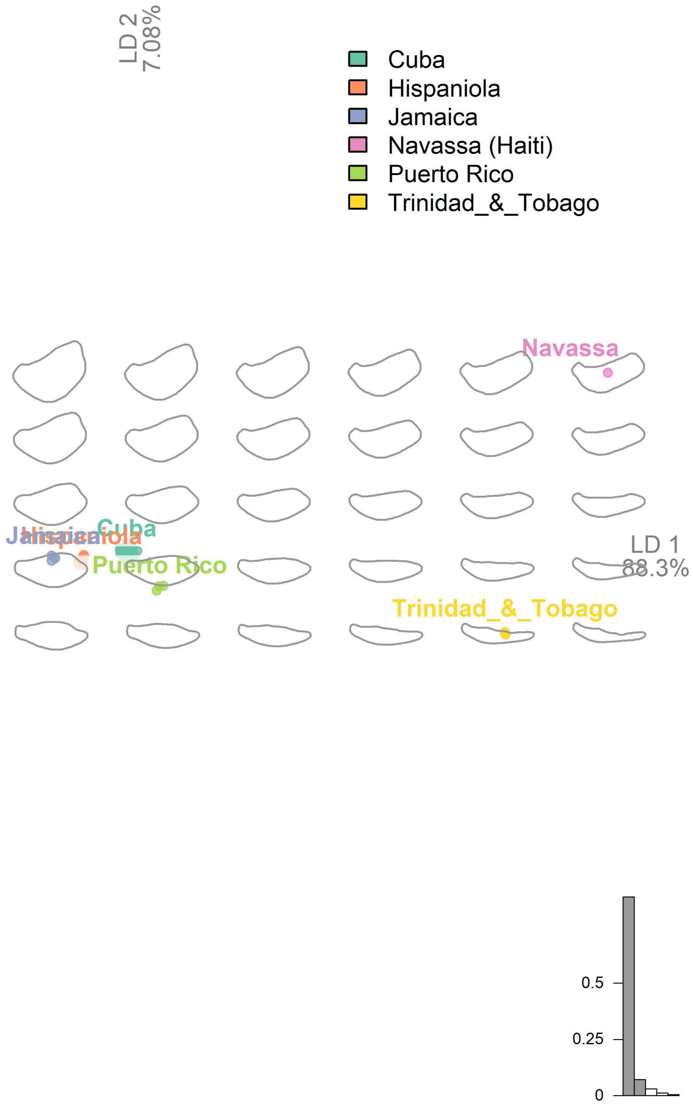

lateral / Caribbean / island

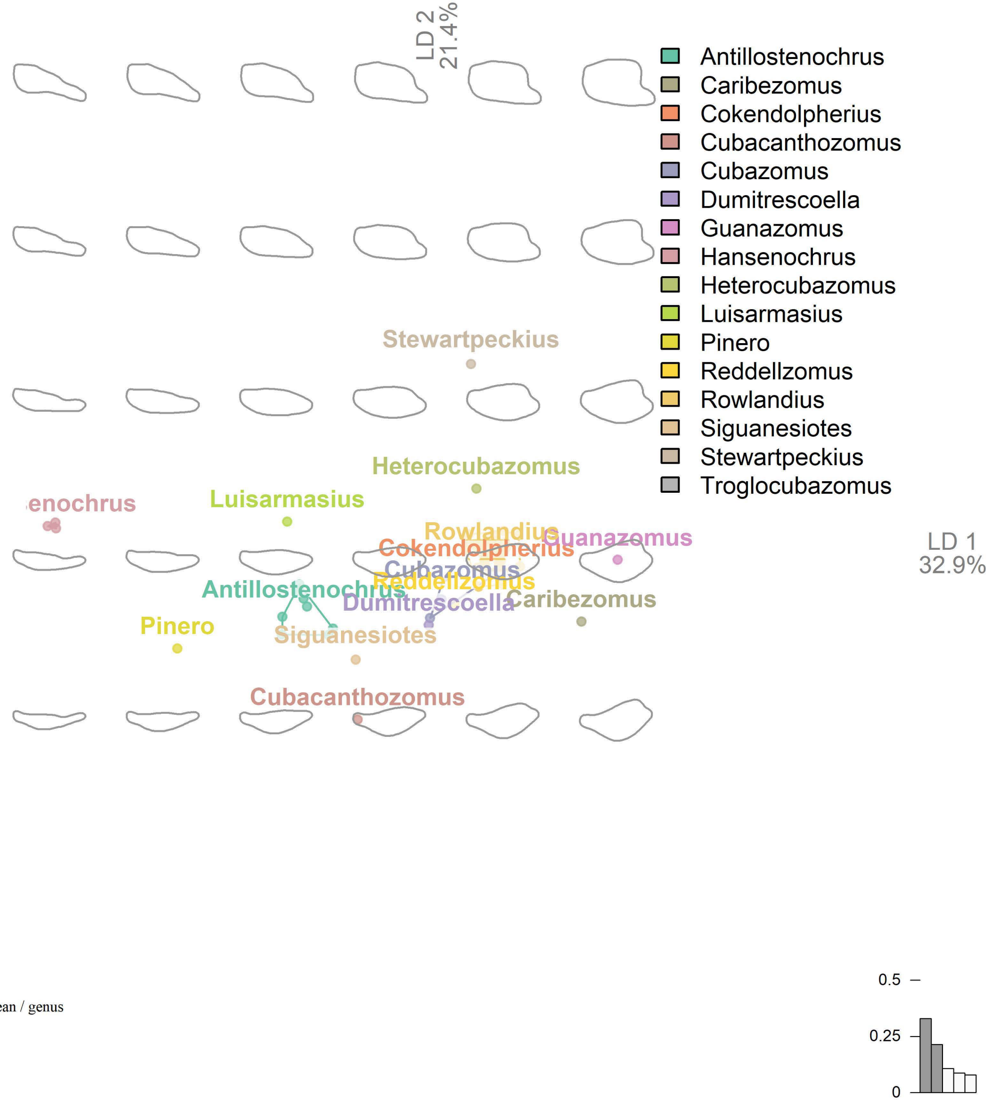

lateral / Caribbean / genus

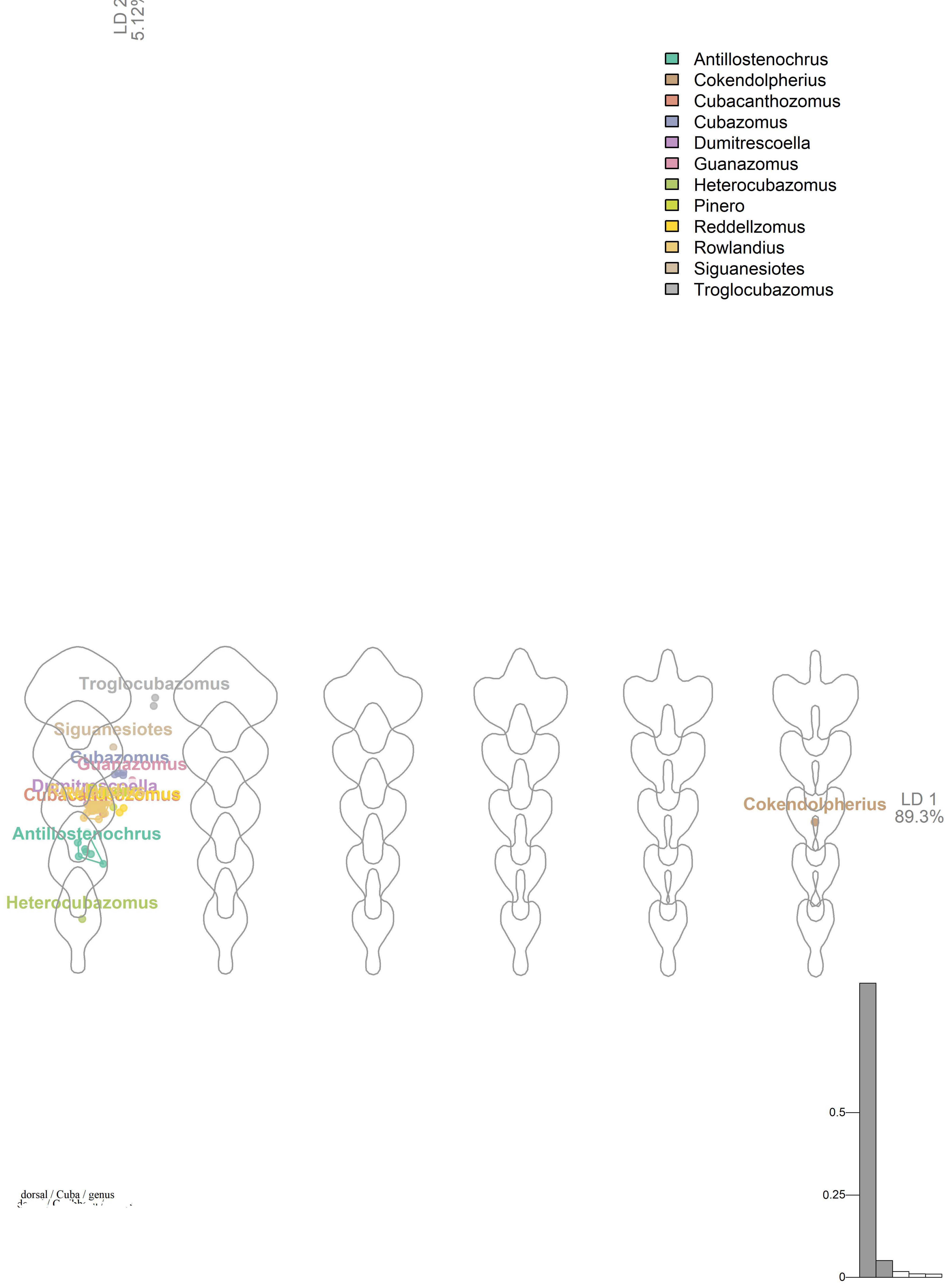
LD 2
5.12%
Antillostenochrus
Cokendolpherius
Cubacanthozomus
Cubazomus
Dumitrescoella
Guanazomus
Heterocubazomus
Pinero
Reddellzomus
Rowlandius
Siguanesiotes
Troglocubazomus
Troglocubazomus
Siguanesiotes
Antillostenochrus
Heterocubazomus
Cokendolpherius
LD 1
89.3%
0.5
0.25
0
dorsal / Cuba / genus

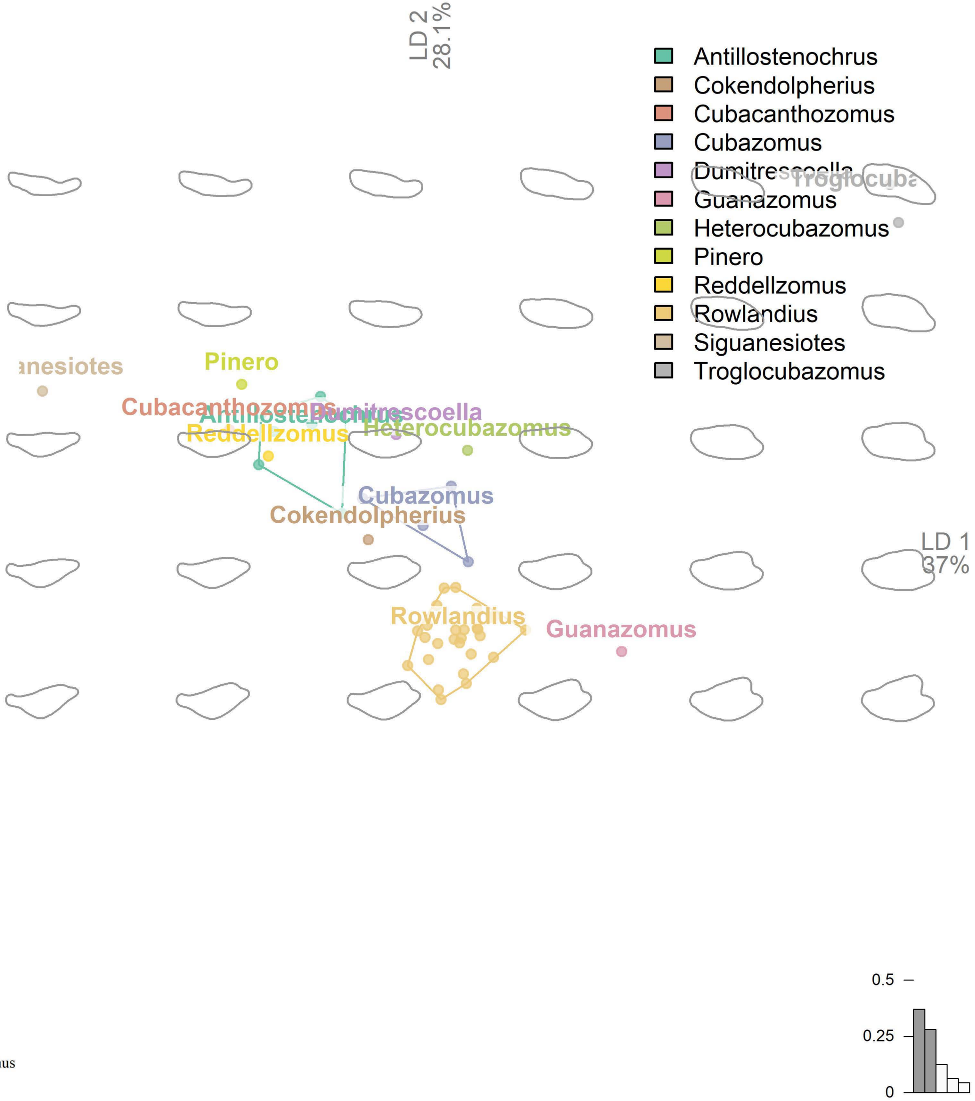

lateral / Cuba / genus
